# The Role of the *Msh2* Mismatch Repair Gene in the *Prdm9*-Driven Hybrid Male Sterility in the House Mouse

**DOI:** 10.3390/genes17070795

**Published:** 2026-07-12

**Authors:** Karel Fusek, Petr Jansa, Jiri Forejt

**Affiliations:** 1 Department of Cell Biology, Faculty of Science, Charles University, 128 00 Prague, Czech Republic; karel.fusek@img.cas.cz; 2 Laboratory of Mouse Molecular Genetics, Institute of Molecular Genetics, Czech Academy of Sciences, 142 20 Prague, Czech Republic; pjansa@img.cas.cz; 3 Laboratory of Epigenetic Regulations, Institute of Molecular Genetics, Czech Academy of Sciences, 142 20 Prague, Czech Republic

**Keywords:** hybrid male sterility, *Msh2*, *Prdm9*, *Mir465*, mismatch repair, meiosis, Dobzhansky–Muller incompatibility, anti-recombination, *Mus musculus*, pachytene arrest

## Abstract

Background/Objectives: Hybrid sterility arises when two fully fertile populations produce sterile offspring, representing a key postzygotic barrier to gene flow between emerging species. In the sterile hybrid males of *Mus musculus domesticus* (*M. m. domesticus*, represented by the C57BL/6J strain, hereafter B6) and *Mus musculus musculus* (*M. m. musculus*, represented by the wild-derived PWD/Ph strain), meiotic prophase I exhibits extensive autosomal asynapsis, pachytene arrest, and an absence of mature spermatozoa. This sterility results from *Prdm9* allelic incompatibility, which is modulated by the X-linked *Hstx2* locus, recently identified as the *Mir465* microRNA. While this two-locus incompatibility is the major cause of sterility, additional modifiers may further contribute to the phenotype. In particular, the DNA mismatch repair protein MSH2 recognizes DNA sequence heterology and enforces anti-recombination in yeast. This raises the possibility that *Msh2* could amplify the effects of subspecific sequence divergence in mouse hybrids. Methods: To test this hypothesis, we generated an *Msh2* knockout on a B6 genetic background and assessed its effect on the hybrid sterility phenotype in F1 hybrid and backcross males. Results: Loss of MSH2 did not restore fertility in F1 hybrids. Testes weight remained low, and no mature spermatozoa were detected. In contrast, a modest partial rescue was observed in backcross males carrying the critical *Prdm9/Mir465* genotype, with improvements in testes weight and sperm count. Conclusions: We examined the role of the *Msh2* gene in arresting spermatogenesis in intersubspecific mouse hybrids. The results show that sterility is predominantly governed by the *Prdm9*-*Mir465* incompatibility, and that DNA mismatch repair has a minor, genetic background-dependent effect.

## 1. Introduction

Hybrid male sterility is a common reproductive barrier that contributes to speciation by restricting gene flow between diverging populations. In the house mouse, hybrid sterility has been studied extensively using the cross between the wild-derived inbred strain PWD/Ph, representing *M. m. musculus*, and the laboratory strain C57BL/6J, representing *M. m. domesticus*. Males from the (PWD × B6)F1 cross exhibit severe meiotic defects, including impaired chromosome pairing and synapsis, which interfere with the initiation and repair of meiotic recombination and ultimately result in pachytene arrest and apoptosis of primary spermatocytes [1]. The genetic basis of this sterility follows the Dobzhansky–Muller model of hybrid incompatibility, in which epistatic interactions between loci that have diverged independently in each subspecies disrupt normal meiotic progression when brought together in the hybrid genome [2,3].

The critical genetic determinant of this sterility is the interaction between the histone methyltransferase *Prdm9* [4], which specifies the positions of meiotic recombination hotspots, and the X-linked *Hstx2* locus [5,6]. Allelic divergence in *Prdm9* between the two subspecies drives widespread autosomal asynapsis in hybrid males. The severity of this meiotic arrest scales with the number of heterosubspecific autosomal pairs in the hybrid genome [6,7]. As few as two such pairs within an otherwise pure *M. m*. *domesticus* genome are sufficient to partially activate this incompatibility [8]. Recent research from our laboratory has identified the *Mir465* microRNA gene cluster as the key hybrid sterility factor within the X-linked *Hstx2* locus [9]. The *Mir465* cluster exhibits copy number variation between the two subspecies, with *M. m. musculus* carrying approximately twice as many copies as *M. m. domesticus*. Deletion of the *Mir465* cluster in hybrid males restores meiotic progression, improves chromosome synapsis, increases the global recombination rate, and rescues fertility in a copy number-dependent manner. The underdominant interaction between *Prdm9* and *Mir465* thus represents the first example of Dobzhansky–Muller incompatibility in hybrids of closely related subspecies of the house mouse.

Beyond these locus-specific incompatibilities, evidence from diverse organisms suggests that conserved meiotic surveillance mechanisms may further modulate hybrid sterility. DNA mismatch repair, and particularly the MSH2 protein, suppresses recombination between diverged homeologous sequences across all domains of life and has been proposed to act as an additional barrier to reproductive isolation. In prokaryotes, specifically in *Escherichia coli*, the MMR system blocks homeologous recombination in intergeneric crosses. Inactivation of MutS, the bacterial ortholog of MSH2, increases recombination 100- to 1000-fold and effectively relaxes the species barrier [10,11,12]. This anti-recombination function is conserved in eukaryotes. In budding yeast hybrids between *Saccharomyces cerevisiae* and *Saccharomyces paradoxus*, which share approximately 12% sequence divergence, meiotic recombination is impaired, and hybrids are nearly sterile. Deletion of *MSH2* in these hybrids restores recombination and dramatically improves fertility, with meiotic-specific repression of *MSH2* and the *SGS1* helicase increasing spore viability up to 70-fold [13,14,15]. Recent research in intraspecies budding yeast hybrids between *S. cerevisiae* strains sharing approximately 2% sequence divergence has further shown that homologous recombination proteins, including Rad51, Rad54 and members of the ZMM complex, themselves antagonize MSH2-containing complexes during meiosis, indicating that the balance between mismatch base pair surveillance and recombination is mutually regulated [16,17].

In *Arabidopsis thaliana*, the role of MSH2 depends on the genetic context. In standard divergent hybrids between accessions Col and Ler, *msh2* mutants show no meiotic defects, unchanged bivalent counts, and high pollen viability, indicating that MSH2 loss neither causes nor rescues sterility in this background [18]. However, in hybrids where interfering Class I crossovers are blocked by *fancm zip4* mutations, sterility becomes severe. In this context, additional *msh2* knockout partially rescues fertility by relieving the suppression of non-interfering Class II crossovers [19].

In mammals, the MutSα complex (MSH2–MSH6) recognizes base pair mismatches in DNA heteroduplex structures, as demonstrated in biochemical and cellular systems [20], and also participates in the surveillance of meiotic recombination intermediates in mice. In mouse intrasubspecific hybrids between the A/J and DBA/2J strains, both of which belong to the *M. m. domesticus* subspecies, loss of MSH2 does not significantly alter crossover frequencies but leads to retention of heteroduplex DNA in recombination products. This indicates that the primary role of MSH2 in mouse meiosis is mismatch correction rather than strong suppression of recombination [21]. However, in the highly polymorphic intersubspecific hybrids of *M. m. musculus* and *M. m*. *domesticus*, where genome-wide divergence reaches approximately 0.92 SNPs per 100 bp together with millions of indels [22,23], males exhibit complete sterility with widespread asynapsis [1,5]. Whether MSH2-mediated mismatch recognition contributes to the recombination failure and synaptic defects in this high-divergence background has not been tested until now.

We hypothesized that meiotic failure in (PWD × B6)F1 hybrid males is amplified by a sequence-dependent anti-recombination checkpoint acting at recombination intermediates. The asymmetry of PRDM9-defined recombination hotspots biases [24] DNA double-strand break (DSB) formation toward one homologue, forcing repair to proceed using a template that frequently contains dense polymorphisms within the heteroduplex DNA formed during strand invasion and joint-molecule maturation. Recognition of these mismatches by MSH2 could destabilize recombination intermediates, suppress crossover completion, and promote persistent unrepaired DSBs that lead to chromosomal asynapsis. In this framework, the critical barrier does not lie primarily in the diverged alleles of sterility genes per se, but in the interaction between diverged hotspot-associated sequence tracts recognized by the conserved DNA mismatch surveillance machinery that interprets such divergence as non-homology.

To test this hypothesis, we used CRISPR/Cas9 to generate *Msh2* null mutants in the B6 strain, then the null allele of *Msh2* was introduced to the PWD background by a series of backcrosses. We then evaluated fertility parameters and meiotic progression in both F1 sterile hybrids and BC1 specific hybrid males. Contrary to our prediction, *Msh2* ablation failed to rescue fertility in F1 hybrids, which remained completely sterile. However, we observed a modest but significant rescue in BC1 males carrying the sterile allelic combination. These results demonstrate that hybrid sterility in this model is primarily driven by the *Prdm9*-*Hstx2*/*Mir465* incompatibility and that MSH2-mediated anti-recombination represents only a minor, genotype-dependent modulatory effect that cannot overcome the primary meiotic barrier in the F1 generation.

## 2. Materials and Methods

### 2.1. Mouse Strains and Maintenance

The inbred laboratory strain C57BL/6J (B6), derived from *M. m. domesticus*, was obtained from The Jackson Laboratory (Bar Harbor, ME, USA). The PWD/Ph (Prague Wild D) strain, derived from *M. m. musculus*, was established and maintained by inbreeding at the Institute of Molecular Genetics of the Czech Academy of Sciences (IMG CAS, Prague, Czech Republic). All animals were maintained under specific pathogen-free (SPF) conditions at the Czech Centre for Phenogenomics (CCP), IMG CAS, BIOCEV, Vestec. All animal experiments were conducted in accordance with Czech legislation on the protection of animals against cruelty (Act No. 246/1992 Coll.; Decree No. 207/2004 Coll.) and were approved by the Ethics Committee of the Institute of Molecular Genetics (Permit No. 45/2020; approval date: 5 May 2020).

### 2.2. Generation of the Msh2 Null Mutant Line

The *Msh2*-knockout line was generated on the C57BL/6J background by CRISPR/Cas9 zygote electroporation at the Transgenic Unit of the Czech Centre for Phenogenomics, IMG CAS. Three sgRNAs targeting exon 7 of *Msh2* were delivered together with recombinant SpCas9 protein as ribonucleoprotein complexes. The founder carried a 29 bp frameshift deletion in exon 7 in a mosaic form. This allele was confirmed by Sanger sequencing, established by germline transmission, and bred to homozygosity. Absence of MSH2 protein in homozygous animals was confirmed by Western blotting. Direct genome editing in PWD/Ph embryos was attempted but failed to establish germline transmission; the *Msh2* null allele was instead transferred onto the PWD background by serial backcrossing (see Transfer of the *Msh2* null allele to the PWD/Ph background). Detailed CRISPR design and breeding protocols are provided in Methods S1.

### 2.3. Genotyping

For routine genotyping, crude genomic DNA was extracted from tail-tip fragments by alkaline lysis (50 mM NaOH at 95 °C for 1.5 h, neutralized with 1 M Tris-HCl pH 8.0). One microliter of crude lysate was used per 16 µL PCR with DreamTaq DNA Polymerase (Thermo Scientific, Waltham, MA, USA, cat. no. EP0701) on a Bio-Rad T100 thermal cycler (Bio-Rad Laboratories, Hercules, CA, USA). *Msh2* alleles (wild-type and the CRISPR-induced 29 bp deletion) were resolved on 2% agarose gels; the SSLP markers M334 (*Prdm9*-linked, Chr 17) and SR51 (*Hstx2*-linked, Chr X) were resolved on 4% agarose gels; the *Prdm9* exon 12 amplicon used for Sanger sequencing of parental and F1 genotypes was resolved on 1.5% agarose gels. All gels were run in 0.5× TBE buffer, stained with ethidium bromide, and visualized under UV illumination alongside wild-type and deletion controls. Three cycling programmes are described in Methods S1. Primer sequences, annealing temperatures, expected amplicon sizes, and references are listed in Table S1.

### 2.4. Sanger Sequencing

Genomic DNA for Sanger sequencing was isolated from tail-tip or spleen tissue using the Puregene Core Kit A (Qiagen, Hilden, Germany, cat. no. 158267) and adjusted to 30 ng/µL. Target loci were amplified by PCR using primers listed in Table S1; amplicons were resolved on 2% agarose gels, excised, and purified using the MinElute Gel Extraction Kit (Qiagen, cat. no. 28606). Purified amplicons were submitted bidirectionally to the SeQme sequencing service (Dobříš, Czech Republic) with 25 pmol of each primer per reaction. Chromatograms were aligned to the C57BL/6J reference genome (GRCm39) in BioEdit (v7.2) and verified by NCBI BLAST (https://blast.ncbi.nlm.nih.gov/). Sanger sequencing was used to confirm the CRISPR-induced 29 bp deletion in the *Msh2*-knockout founder and subsequent generations, and to verify *Prdm9* alleles in parental strains, F1 hybrids, and B6-*Msh2* founders entering the BC1 cross. Detailed reagent compositions and the complete sequencing workflow are provided in Methods S1.

### 2.5. Western Blotting

Western blotting on whole testis lysates was used to confirm the absence of MSH2 protein in knockout animals. Testes were homogenized in ice-cold RIPA lysis buffer (50 mM Tris-HCl pH 7.4, 150 mM NaCl, 1% NP-40, 0.5% sodium deoxycholate, 0.1% SDS) supplemented with complete EDTA-free protease inhibitor cocktail (Roche, Mannheim, Germany, cat. no. 11873580001). Lysates were cleared by centrifugation at 14,000× *g* for 15 min at 4 °C; protein concentration was determined by BCA assay (Pierce, Thermo Fisher Scientific, Rockford, IL, USA, cat. no. 23225). Equal amounts of protein (30 µg per lane) were separated by SDS-PAGE on 4–12% Bis-Tris gels under MOPS buffer and transferred to PVDF membranes (Merck Millipore, Burlington, MA, USA, cat. no. IPVH00010). Membranes were blocked in 5% non-fat dry milk in TBS-T (0.1% Tween-20) for 1 h at room temperature, then probed overnight at 4 °C with rabbit monoclonal anti-MSH2 antibody (Abcam, Cambridge, UK, ab212188, clone EPR21017-2, 1:1 000) and mouse monoclonal anti-β-actin antibody (Abcam ab8226, 1:5 000). HRP-conjugated secondary antibodies (Abcam ab205718 anti-rabbit and ab205719 anti-mouse, each 1:20,000) were applied for 1 h at room temperature. Signal was detected with Abcam ECL Western blotting Substrate (ab133406) on a ChemiDoc imaging system (Bio-Rad Laboratories, Hercules, CA, USA). Antibody details are provided in Table S2.

### 2.6. Transfer of the Msh2 Null Allele to the PWD/Ph Background

Because crossing PWD females with B6 males produces sterile male hybrids, the *Msh2* null allele was transferred onto the PWD background through successive backcross generations using fertile heterozygous female carriers. A B6-*Msh2*^−/−^ male was first crossed with a wild-type PWD female. Heterozygous female offspring (PWD.B6–*Msh2*^+/−^) were selected by genotyping at each generation and mated with wild-type PWD males. By generation N5, progeny carry approximately 96.9% PWD genome (sub-congenic); from N10 onward, the line is considered fully congenic (>99.9% PWD). Animals used in this study were derived from generations N6–N10 and are referred to as PWD.B6ᴺ–*Msh2*^+/−^ throughout.

### 2.7. Backcross Strategy for Assessing Msh2 Ablation in Hybrid Males

To test the effect of *Msh2* deficiency in a hybrid genetic context before full congenic status was achieved, a BC1 backcross scheme [(B6-*Msh2*^+/−^ × PWD) × B6-*Msh2*^+/−^] was employed. A B6-*Msh2*^+/−^ female was crossed with a wild-type PWD male to generate heterozygous F1 females, which were then crossed with B6-*Msh2*^+/−^ males to produce the BC1 generation (hereafter (BxP) × B). BC1 males segregate for *Msh2* (wt/wt, wt/KO, KO/KO), *Prdm9* (B6/B6 or B6/PWD), and *Hstx2* (B6 or PWD allele), carrying on average 75% B6 and 25% PWD autosomal genome. All males were genotyped at all three loci and assigned to experimental groups accordingly. BC1 males were genotyped using primer pairs listed in Table S1: the *Msh2* F2/R2 and F3/R3 pairs to discriminate the wild-type and 29 bp deletion alleles, the M334 SSLP marker as a surrogate for the *Prdm9* allele on Chr 17, and the SR51 SSLP marker as a surrogate for the *Hstx2* allele on Chr X.

### 2.8. Fertility Parameters

Testes weight, relative testes weight (rTW = testes weight/body weight), and epididymal sperm count were measured in sexually mature males aged 10–15 weeks. Both epididymides were dissected into 1 mL of 1× PBS, minced, and shaken to release spermatozoa. After 1 min on ice, an aliquot was taken from the central portion of the suspension and diluted according to expected sperm density (1:50–1:100 for fertile males, 1:20–1:30 for males with reduced testes weight, undiluted for sterile males). A 10 µL aliquot was loaded onto a Bürker counting chamber (Meopta, Přerov, Czech Republic), and spermatozoa were counted in 25 central squares in duplicate. Total sperm concentration per millilitre was calculated as *N* = D × *n̄*_25_ × 10^4^, where D is the dilution factor, and *n̄*_25_ is the mean count across both chambers. Per-male body weight, testes weight, relative testes weight, and sperm count for all measured males (B6-*Msh2*, BC1, F1) are provided in Table S3.

### 2.9. Meiotic Chromosome Spreads and Immunofluorescence

Spermatocyte spreads were prepared from freshly dissected testes as described previously [1,6]. Briefly, seminiferous tubules were released into cold RPMI 1640 (Sigma, St. Louis, MO, USA, cat. no. R8758), dissociated mechanically, filtered through a 40 µm cell strainer (Corning, Corning, NY, USA, cat. no. 352340) and resuspended in 0.1 M sucrose containing protease inhibitors. After hypotonic treatment on ice for 13 min, the suspension was spread onto slides pre-coated with 1% paraformaldehyde and incubated in a humidified chamber at 4 °C for 3 h. Slides were washed, air-dried, and blocked with 0.5× MAH buffer (1.5% BSA, 5% goat serum, 0.05% Triton X-100) for 4h at 4 °C. Primary antibodies (Table S2) were applied overnight at 4 °C in 0.5× MAH buffer. The following combinations were used: (i) HORMAD2, SYCP3, and centromere marker to assess autosomal asynapsis; (ii) MLH1 or MLH3, SYCP1, and centromere marker to quantify crossover sites; (iii) DMC1, SYCP3, and H1t to monitor DSB formation and repair across prophase I substages; and (iv) γH2AX and SYCP3 to assess persistence of DSB-associated chromatin modifications. After washing, fluorophore-conjugated secondary antibodies (Table S2) were applied for 2–3 h at 4 °C. Slides were mounted with Vectashield containing DAPI (Vector Laboratories, Newark, CA, USA, cat. no. H-1200). Images were acquired on a Nikon Eclipse 400 epifluorescence microscope (Nikon Corporation, Tokyo, Japan) with a 60× Plan Fluor objective and a DS-QiMc CCD camera and processed using NIS-Elements software (Nikon, Tokyo, Japan, version 4.20.03). A minimum of 50 nuclei per individual were scored for each marker combination. Per-male and per-cell cytology data are provided in Table S4 (B6-*Msh2* control cohort) and Table S5 (BC1 cohort).

### 2.10. Validation of Genomic Background Composition in BC1 Males by MiniMUGA

To verify the expected autosomal genomic composition in BC1 males, a subset of individuals carrying the sterile allelic combination (*Prdm9^B6/PWD,^ Hstx2^PWD^*) was genotyped using the MiniMUGA array [25] (Neogen Corporation, The Dairy School, South Ayrshire, UK). This platform provides genome-wide SNP coverage across all autosomes and the X chromosome and allows chromosome-level assessment of B6/PWD genomic content in individual males. Raw genotype calls were processed through a five-step marker quality filter (parental control discordance, F1 heterozygosity check, B6-*Msh2* background check, X-chromosome hemizygosity, BC1 singleton check), removing 346 markers and retaining 3 270 informative markers for downstream analysis. The filtered data were used to confirm that BC1 males carry approximately 75% B6 and 25% PWD autosomal genome and to visualize the distribution of subspecific segments across individual chromosomes. Detailed MiniMUGA methodology, including the five-step quality filter, is provided in Methods S1. Per-male marker calls and the filter trace are provided in Table S6.

### 2.11. Statistical Analysis

All statistical analyses were performed in Python (v3.11) using the scipy.stats and statsmodels modules. Normality within each group was assessed using the Shapiro–Wilk test, and because several groups violated normality, non-parametric tests were used for the fertility endpoints [26]. Pairwise comparisons between *Msh2* genotypes used two-sided Mann–Whitney *U* tests [27], monotonic trends across the genotype gradient (wt → KO/wt → KO/KO) were tested with the Jonckheere–Terpstra trend test [28], and azoospermia outcomes were compared with two-sided Fisher’s exact tests. Spearman rank correlation assessed associations between autosomal asynapsis (HORMAD2) and fertility parameters on a per-male basis. Recombination intermediate counts (DMC1, MLH3) were analyzed by linear mixed models with a random intercept per male [29]. The Benjamini–Hochberg false discovery rate procedure was applied within each cohort across sperm count and relative testes weight as endpoints [30]: 9 tests in B6-*Msh2*, 24 pairwise and 8 trend tests in BC1, and 6 pairwise and 2 trend tests in F1 hybrids. Absolute testes weight and azoospermia were reported descriptively without correction. Data are reported as median [IQR]. Detailed methods are provided in Methods S1 and Statistics S1.

## 3. Results

### 3.1. Genetic Strategy for Evaluating Msh2 in Intersubspecific Hybrid Sterility

**Figure 1:**
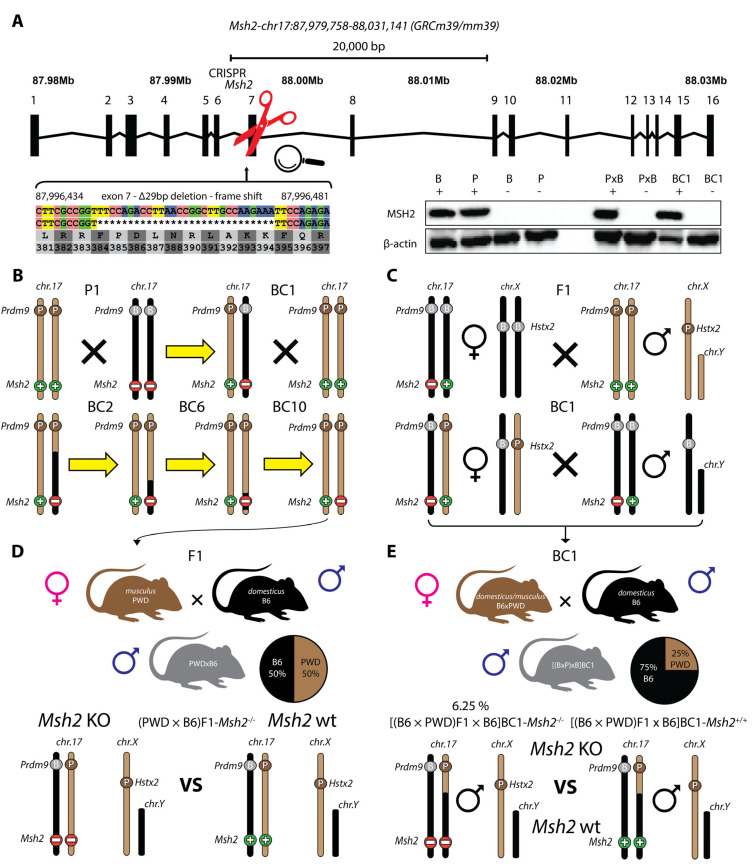
**Genetic strategy to evaluate the impact of *Msh2* deficiency in *Prdm9*-driven hybrid male sterility.** (**A**) CRISPR/Cas9-mediated generation and validation of the *Msh2* null allele on the B6 background. Top: genomic structure of *Msh2* (chr17:87,979,758-88,031,141, GRCm39) with CRISPR target site at exon 7. Left: identification by Sanger sequencing of the 29 bp deletion in exon 7 introducing a frameshift mutation. Right: Western blotting confirming absence of MSH2 protein in homozygous knockouts across B6 (B), PWD (P), F1 (P × B) and BC1 backgrounds with beta-actin loading control. (**B**) Backcrossing strategy to transfer the *Msh2* KO allele into the PWD background. Chromosome 17, which carries both the *Prdm9* and *Msh2* genes, is shown here. Brown and black indicate the allele derived from the PWD or B6 strain, respectively. The *Msh2* alleles are shown as red minus for *Msh2* KO and green plus for *Msh2* wt. The null allele derived from the B6 line was introgressed through successive generations of backcrossing with the PWD line, resulting in the PWD-*Msh2*^+/−^ congenic mice. (**C**) BC1 experimental design. B6-*Msh2*^+/−^ females were crossed with PWD males to generate F1 hybrids. F1 females carrying the *Msh2* KO allele and the PWD X chromosome (*Hstx2^PWD^*) were then backcrossed with B6 males, producing BC1 males. BC1 males segregated independently for the *Msh2* genotype (KO/KO vs. wt) and *Prdm9* genotype (B6/PWD het vs. B6/B6), while all carrying the *Hstx2^PWD^* allele. On average, BC1 males carry ~75% B6 and ~25% PWD autosomal genomic content. (**D**) Left: canonical (PWD × B6) F1 hybrid. Sterile males—causing allelic combination context (*Prdm9^B6/PWD^*, *Hstx2^PWD^*—PB/P), *Msh2*^−/−^ vs. *Msh2*^+/+^ males. The genetic background of both strains is represented in the 50:50 (B/P) ratio. (**E**) Right: BC semi-sterile males representing allelic combination (*Prdm9^B6/PWD^*, *Hstx2^PWD^*—PB/P), *Msh2^−/−^* vs. *Msh2*^+/+^ males. The genetic background of both strains is represented in a random mix 75:25 (B/P) ratio.

To determine whether loss of *Msh2*-driven mismatch repair can modify hybrid male sterility caused by the *Prdm9*-*Hstx2*/*Mir465* incompatibility, we first generated and validated a null allele of *Msh2* on the C57BL/6J background using CRISPR/Cas9 and characterized the fertility phenotype of B6-*Msh2*^−/−^ males (Figure 1A). This was an essential step to separate the intrinsic effects of *Msh2* loss on spermatogenesis from any hybrid-specific interactions. To produce the canonical (PWD × B6) F1 hybrids (Figure 1D) carrying the sterility-causing combination of *Prdm9*^B6/PWD^ and *Hstx2*^PWD^, together with *Msh2*^−/−^, we had to introduce the knockout allele onto the PWD background. Direct genome editing in PWD embryos failed repeatedly due to the high vulnerability of PWD oocytes. We therefore transferred the B6-derived null allele to the PWD background by serial backcrossing. After five generations (N5), the line was considered sub-congenic, whereas after ten generations (N10), it was regarded as fully congenic, with the genome being predominantly of PWD origin except for the introgressed *Msh2* locus (Figure 1B).

While the backcrossing to PWD background was in progress, we generated in parallel the [(B6 × PWD) × B6] backcross (BC1) males that enabled us to test the effect of *Msh2* ablation in a hybrid genetic context before full congenic status was achieved. In the BC1 (Figure 1C) generation, males segregated independently for *Msh2* genotype, *Prdm9* allele combination, and *Hstx2* allele. As a result, the phenotype depended on the specific combination of *Prdm9* and *Hstx2* alleles, with only a specific allelic combination (*Prdm9^B6^*^/*PWD*^–*Hstx2^PWD^*) causing hybrid male sterility (Figure 1E). Because these males carry on average 75% of the B6 genome and 25% of the PWD genome, the sterility phenotype is attenuated compared to F1 hybrids [7], providing a dynamic range in which a partial rescue might be detectable.

### 3.2. Loss of Msh2 Impairs Spermatogenesis on the Pure B6 Background

**Figure 2:**
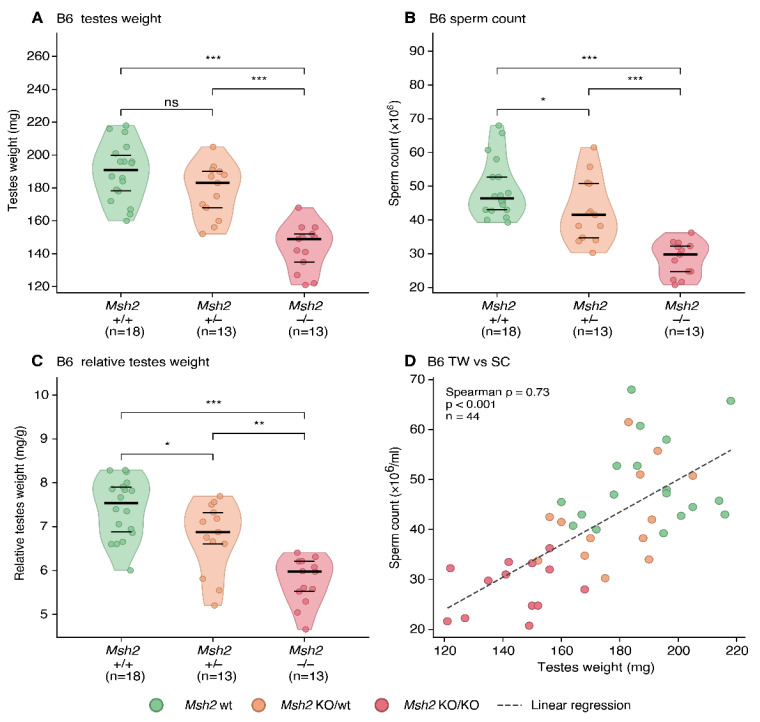
***Msh2* deficiency impairs spermatogenesis on the C57BL/6J background.** Reproductive parameters of *Msh2*^+/+^, *Msh2*^+/−^ and *Msh2*^−/−^ males on a pure C57BL/6J background (*n* = 18, 13, 13). (**A**) Testes weight (mg). Individual data points are shown as a jittered strip plot overlaid on a violin and box-and-whisker plot, with each genotype in a distinct colour (*Msh2*^+/+^: green; *Msh2*^+/−^: orange; *Msh2*^−/−^: pink/red). Violins show kernel density; the thick horizontal bar marks the median and the two thin bars the 25th and 75th percentiles (interquartile range, IQR). Significance brackets show pairwise comparisons (two-sided Mann–Whitney *U* test with Benjamini–Hochberg FDR correction across all nine B6 pairwise comparisons, three *Msh2* pairs × three endpoints). (**B**) Sperm count (×10^6^ per paired epididymis) in the same males. Plot layout and statistical notation as in (**A**). (**C**) Relative testes weight (testes weight/body weight, mg g^−1^) in the same males. Plot layout and statistical notation as in (**A**). (**D**) Scatter of testes weight versus sperm count across all 44 males, coloured by *Msh2* genotype as in (**A**). The solid line shows the ordinary least-squares regression fit (Spearman *ρ* = 0.73, ***, *n* = 44). Significance thresholds: * *p* < 0.05, ** *p* < 0.01, *** *p* < 0.001; ns, not significant.

Before examining the effects of *Msh2* ablation in the hybrid context, we characterized the fertility phenotype of *Msh2*^−/−^ on the pure B6 background. Adult B6-*Msh2*^−/−^ males showed a significant reduction in absolute testes weight (weight of paired testes in milligrams) compared to wild-type controls (143.8 ± 14.2 mg vs. 189.7 ± 17.6 mg, *p* < 0.001, *n* = 13 and 18) (Figure 2A). Relative testes weight (in milligrams per body weight in grams) was similarly decreased (5.76 ± 0.53 vs. 7.41 ± 0.68 mg/g, *p* < 0.001), confirming that the difference was not attributable to variations in body mass (Figure 2C). Epididymal sperm counts were also significantly reduced in knockout males (28.5 ± 5.1 vs. 49.2 ± 8.7 × 10^6^, *p* < 0.001). Testes weight and sperm count were positively correlated across individuals (r = 0.70, *p* < 0.001, *n* = 44) (Figure 2D). Per-mouse data are provided in Table S3 and Statistics S1. B6-*Msh2*^+/−^ males showed no significant reduction in testes weight (BH-adjusted *p* = 0.101) (Figure 2A) but displayed small yet statistically significant reductions in relative testes weight (BH-adjusted *p* = 0.044) and epididymal sperm count (BH-adjusted *p* = 0.041) (Figure 2B). The associated effect sizes were modest (rank-biserial |r| ≈ 0.36–0.46), and heterozygous males remained overtly fertile, indicating that a single functional copy of *Msh2* is sufficient to maintain normal spermatogenesis.

We also performed cytological analysis of meiotic prophase I in a single individual per genotype. No overt differences were observed in autosomal asynapsis (assessed by HORMAD2 localization) or in crossover frequency (assessed by MLH3 foci counts) between B6-*Msh2*^−/−^ and B6 wild-type spermatocytes (Table S4). However, fewer DMC1 foci, which mark early recombination intermediates, were observed at pachytene in the B6-*Msh2*^−/−^ male compared to the control. Given the single-individual sample size, this observation remains preliminary (Figure S1).

Taken together, these data established two important reference points for the analysis of hybrid males. First, loss of *Msh2* is intrinsically detrimental to spermatogenesis even in the absence of any hybrid incompatibility. Second, this detrimental effect is strictly recessive. Any rescue observed in the *M. m. musculus*/*M. m. domesticus* hybrid context by the *Msh2*^−/−^ knockout would imply that the anti-recombination function of MSH2 contributes to spermatogenesis failure in hybrids.

### 3.3. Msh2 Ablation Partially Rescues Fertility in BC1 Males Carrying the Sterile Allelic Combination

**Figure 3:**
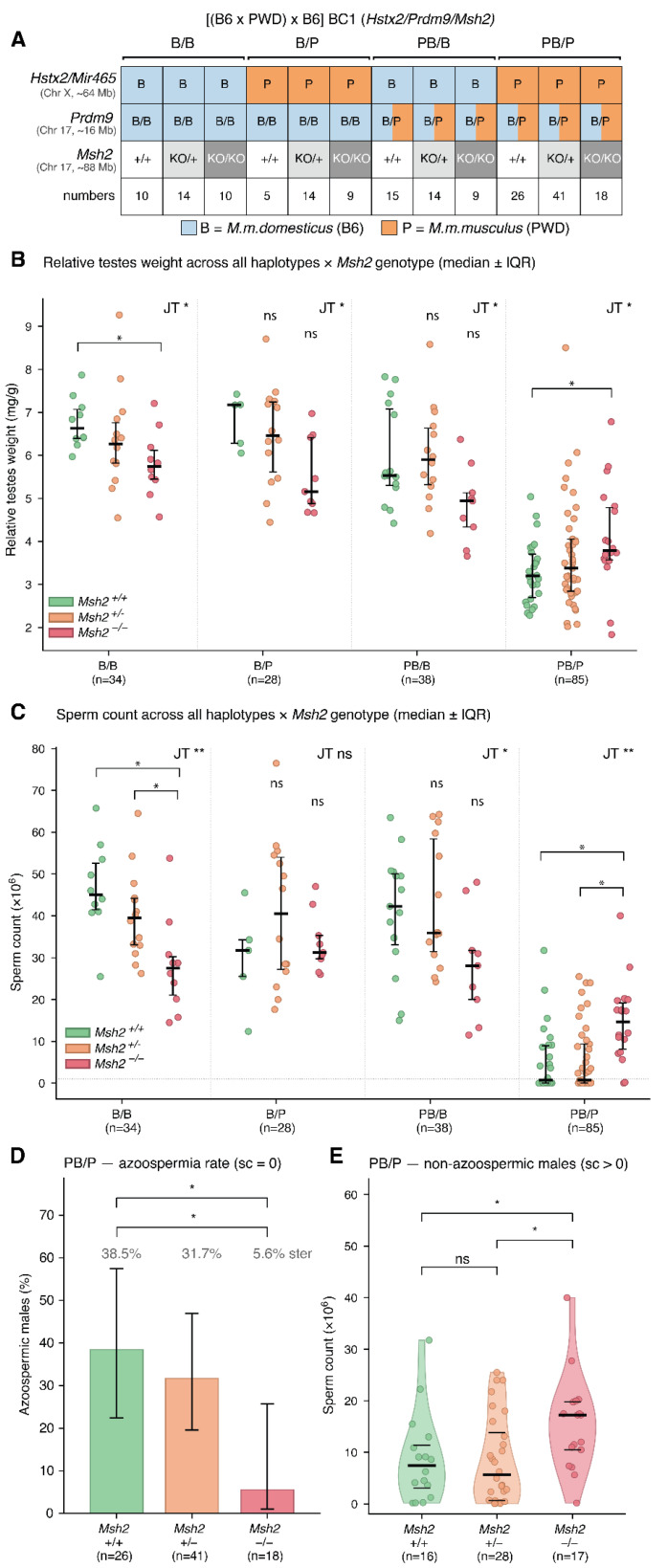
***Msh2* genotype affects fertility parameters of BC1 males in a genotype-dependent manner.** Reproductive parameters of 185 BC1 males (genotype [B6-*Msh2*^+/−^ × PWD] × B6-*Msh2*^+/−^), stratified by *Prdm9*/*Hstx2* allelic combination (B/B, PB/B, B/P, PB/P) and *Msh2* genotype. (**A**) Genotype matrix of the BC1 cohort generated by the [(B6 × PWD) × B6] backcross. Animals are stratified by *Hstx2/Mir465* (Chr X, ~64 Mb), *Prdm9* (Chr 17, ~16 Mb) and *Msh2* (Chr 17, ~88 Mb). Blue (B) marks the *M. m. domesticus* (B6) allele and orange (P) the *M. m. musculus* (PWD) allele. The first symbol of each label refers to *Hstx2/Mir465* and the second to *Prdm9*, yielding four allelic classes (B/B, B/P, PB/B, PB/P). Numbers in the bottom row give animals per *Msh2* genotype within each class. (**B**) Relative testes weight (mg g^−1^) across the four classes split by *Msh2* genotype. Individual data points are shown as a jittered strip plot overlaid on median ± IQR bars; each dot is one male (*Msh2*^+/+^: green; *Msh2*^+/−^: orange; *Msh2*^−/−^: pink/red). The Jonckheere–Terpstra (JT) trend test was applied within each class as the primary test (one-sided, direction set per class from the independent B6-*Msh2* cohort), with Benjamini–Hochberg correction across all 8 tests; adjusted significance is shown above each class. Pairwise Mann–Whitney *U* comparisons are exploratory secondary tests without further correction and are shown as brackets. Sample sizes per class are given on the x-axis. (**C**) Sperm count (×10^6^ per paired epididymis) across the four classes split by *Msh2* genotype. Plot conventions and colour code as in (**B**). On the B/B background, sperm count decreases progressively with loss of *Msh2*. The trend reverses in the PB/P class, where *Msh2*^−/−^ males show higher sperm count than *Msh2*^+/+^ and *Msh2*^+/−^ littermates. B/P shows no significant trend, and PB/B shows an overall trend without significant pairwise differences. (**D**) Azoospermia rate (SC = 0) in PB/P males with 95% Wilson confidence intervals. *Msh2*^+/+^: 38.5% (*n* = 26); *Msh2*^+/−^: 31.7% (*n* = 41); *Msh2*^−/−^: 5.6% (*n* = 18). Pairwise differences tested with Fisher’s exact test: *Msh2*^+/+^ vs. *Msh2*^−/−^ (odds ratio = 10.6, *) and *Msh2*^+/−^ vs. *Msh2*^−/−^ (*). (**E**) Sperm count among non-azoospermic PB/P males only (SC > 0; *n* = 16 *Msh2*^+/+^, 28 *Msh2*^+/−^, 17 *Msh2*^−/−^). Significance thresholds: * *p* (or *q*) < 0.05, ** < 0.01, ns, not significant.

If MSH2-mediated mismatch recognition amplifies the spermatogenesis failure in hybrids, its removal should be beneficial in males carrying the sterile allelic combination *Prdm9*^B6/PWD^ together with *Hstx2*^PWD^ (hereinafter referred to as PB/P). To test this prediction, we used the BC1 backcross, which segregates independently for the three loci and provides all four genotype combinations within a single cohort (Figure 3A).

Among BC1 males with the sterile PB/P genotype, we detected a significant monotonic increase in both sperm output and relative testes weight across the ordered *Msh2* genotype series (Jonckheere–Terpstra trend test, sperm count *p* = 0.009, relative testes weight *p* = 0.028 after FDR correction). BC1-*Msh2* KO/KO males showed significantly higher sperm counts than wild-type controls with the same allelic combination (median 14.6 vs. 0.8 × 10^6^, BH-adjusted *p* = 0.012, *n* = 18 vs. 26) and increased relative testes weight (median 3.79% vs. 3.20%, BH-adjusted *p* = 0.034). The proportion of azoospermic males was markedly reduced in *Msh2* KO/KO compared with wild-type PB/P animals (1/18, 5.6% vs. 10/26, 38.5%; Fisher’s exact test, OR = 10.6, *p* = 0.016), demonstrating that complete loss of MMR function converts a substantial fraction of males from azoospermia to oligospermia in this allelic combination context. Heterozygotes did not differ from wild-type males (*p* > 0.05), indicating that the rescue is strictly recessive and requires complete loss of MMR function (Figure 3B–E).

Strikingly, the effect of *Msh2* genotype was qualitatively opposite in males carrying the fertile allelic combination *Prdm9*^B6/B6^, *Hstx2*^B6^ (hereinafter referred to as B/B). In these males, *Msh2*^−/−^ was associated with reduced sperm output (median 27.5 vs. 45.0 × 10^6^, BH-adjusted *p* = 0.034) and lower relative testes weight (median 5.75 vs. 6.63, BH-adjusted *p* = 0.034) (Figure 3B,C). This mirrors the intrinsic negative effect of *Msh2* loss documented on the pure B6 background (Figure 2A–C), confirming that without hybrid incompatibility, loss of MSH2 impairs rather than enhances spermatogenesis. BC1 males of the B/B allelic combination carry approximately 75% B6 autosomal genome and no PWD-derived sterility alleles, and their response to *Msh2* deficiency is therefore indistinguishable from that observed on the pure B6 background. The two intermediate allelic combinations each carry only one component of the sterile genotype. Males of the allelic combination *Prdm9^B6/B6^*, *Hstx2^PWD^* (hereinafter referred to as B/P) carry the PWD-derived X chromosome without the heterozygous *Prdm9* genotype. In these males, *Msh2* KO/KO did not differ from wild-type in sperm output (median 31.3 vs. 31.8 × 10^6^, BH-adjusted *p* = 0.82), with only a non-significant reduction in relative testes weight (median 5.16 vs. 7.17, BH-adjusted *p* = 0.076) (Figure 3B,C). Males of the combination *Prdm9^B6^*^/PWD^, *Hstx2^B6^* (hereinafter referred as PB/B) carry the heterozygous *Prdm9* genotype without the PWD-derived X. These males showed a non-significant trend toward reduced sperm output (median 28.1 vs. 42.3 × 10^6^, BH-adjusted *p* = 0.076) and reduced relative testes weight (median 4.94 vs. 5.54, BH-adjusted *p* = 0.076) in KO/KO individuals (Figure 3B,C). No comparison between *Msh2* genotypes reached significance after correction for multiple testing in either combination (all BH-adjusted *p* ≥ 0.052). Where present, these trends were directed toward reduced output in KO/KO males, directionally consistent with the intrinsic effect of *Msh2* loss rather than with rescue. This is consistent with the requirement for the PB/P genotype to elicit MSH2-modifiable spermatogenic failure.

The opposing direction of the *Msh2* effect between the fertile and sterile allelic combination provides evidence that MMR-mediated mismatch base pair recognition acts as an additional, genotype-dependent incompatibility layer that amplifies the primary *Prdm9*-*Hstx2*/*Mir465* Dobzhansky–Muller incompatibility.

To examine whether the partial fertility rescue in BC1 *Msh2*^−/−^ PB/P males is reflected at the cytological level, we quantified autosomal asynapsis by HORMAD2/SYCP3 co-staining of pachytene spermatocyte spreads in 16 BC1 PB/P males (4 *Msh2*^+/+^, 6 *Msh2*^+/−^, 6 *Msh2*^−/−^). The frequency of pachytene cells carrying at least one HORMAD2-positive asynapsed autosome decreased monotonically across the *Msh2* allelic series (medians: *Msh2*^+/+^ 46.3%, *Msh2*^+/−^ 27.6%, *Msh2*^−/−^18.1%; Jonckheere–Terpstra one-sided trend test, *p* = 0.030). The pairwise comparison between *Msh2* wt and *Msh2* KO/KO confirmed the directional effect (Mann–Whitney U, one-sided, *p* = 0.057, ns). Per-mouse asynapsis frequency was negatively correlated with testes weight (Spearman ρ = −0.55, *p* = 0.026, *n* = 16) and with sperm count (ρ = −0.44, *p* = 0.086, ns) (Figure S2). Pairwise comparisons between individual *Msh2* genotypes did not reach significance after multiple-testing correction, consistent with a gradual quantitative effect distributed across the allelic series rather than a threshold switch. Per-mouse and per-cell HORMAD2 data are provided in Table S5.

To assess earlier meiotic events, we quantified DMC1 foci as a marker of programmed DSB formation and repair and MLH3 foci as a marker of Class I crossovers in a smaller subset of BC1 PB/P males (DMC1: *Msh2*^+/+^ *n* = 2, *Msh2*^+/−^ *n* = 3, *Msh2*^−/−^ *n* = 2; MLH3: *Msh2*^+/+^ *n* = 2, *Msh2*^+/−^ *n* = 4, *Msh2*^−/−^ *n* = 2). Linear mixed models with mouse identity as random intercept detected a significant increase in total DMC1 foci in *Msh2^−/−^* spermatocytes at zygotene (*p* = 0.0004), with a similar trend at pachytene that did not reach significance (*p* = 0.082). MLH3 focus counts showed a modest increase in *Msh2*^−/−^ relative to *Msh2* wt (+1.9 foci per cell, *p* = 0.008) (Figures S3 and S5). Given the limited number of mice per genotype and the variability in individual fertility within the cytological subset, these analyses are reported as exploratory. Per-mouse and per-cell data are provided in Table S5.

**Figure 4:**
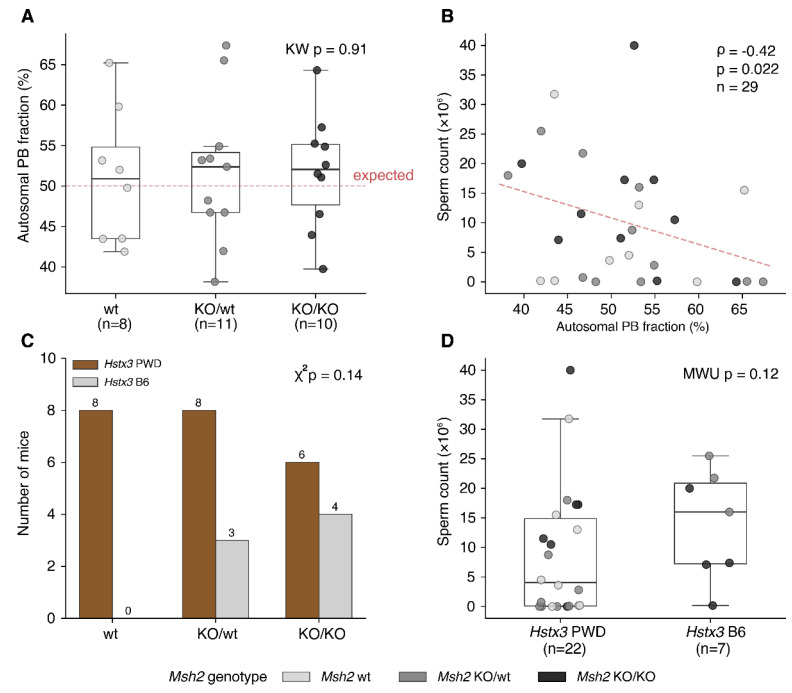
**Analytical validation of genomic background composition and assessment of potential confounding loci in PB/P BC1 males.** (**A**) Distribution of the autosomal PB fraction (percentage of heterozygous B6/PWD markers) in PB/P BC1 males stratified by *Msh2* genotype. Individual data points are shown as a jittered strip plot overlaid on box-and-whisker plots (box: IQR; bar: median; whiskers: 1.5× IQR). The dashed red line indicates the expected 50%. The three groups do not differ significantly (Kruskal–Wallis *p* = 0.91), confirming that the observed fertility differences between *Msh2* genotypes are not attributable to unequal autosomal genomic backgrounds. (**B**) Spearman rank correlation between total autosomal PB fraction and epididymal sperm count across all 29 genotyped PB/P males. Each point represents one individual, coloured by *Msh2* genotype as in panel (**A**). The dashed line shows the ordinary least-squares regression fit. A significant negative correlation was detected (ρ = −0.42, *p* = 0.022), consistent with the established relationship between heterosubspecific autosomal heterozygosity and spermatogenic impairment [7,8]. (**C**) Distribution of the *Hstx3* allele (PWD vs. B6) across *Msh2* genotype groups. The *Hstx3* locus (Chr X, 0–7.23 Mb [8]) segregates as a consequence of maternal recombination in females preselected for the *Hstx2*^PWD^ allele. All eight wt males carry the PWD allele at *Hstx3*, whereas the B6 allele is present only in KO/wt (3/11) and KO/KO (4/10) groups. This distribution does not deviate significantly from random (χ^2^ test, *p* = 0.14) and reflects linkage between *Hstx3* and *Hstx2* on the X chromosome. (**D**) Sperm count in PB/P BC1 males stratified by *Hstx3* allele. Individual data points are coloured by *Msh2* genotype (light grey: wt; mid grey: KO/wt; dark grey: KO/KO). Males carrying the B6 allele at *Hstx3* show nominally higher sperm counts, consistent with the attenuating effect. The absence of wt males in the *Hstx3*^B6^ group precludes separation of the *Hstx3* effect from the *Msh2* rescue effect within this subset.

To validate the genomic background of the BC1 cohort, we genotyped 29 males carrying the sterile PB/P allelic combination together with five parental controls using the MiniMUGA SNP array [25,31]. Reference genotypes from the parental PWD and B6 strains were used to apply a five-step quality filter removing markers with discordant calls in parental strains, F1 hybrid controls, or the B6-*Msh2* control, heterozygous calls in the hemizygous male X chromosome, and isolated singleton genotype calls in BC1 individuals (Methods S1, Table S6). This procedure removed 346 markers, leaving 3270 informative autosomal and X-linked markers. Chromosome-level reconstruction of subspecific genomic composition confirmed the expected BC1 architecture (Figure S4A–C). All autosomes carried either homozygous B6/B6 or heterozygous B6/PWD segments, with recombinant chromosomes reflecting maternal crossovers in the F1 generation. The mean autosomal heterozygosity across individuals was 51.5% of markers, consistent with the expected 50% for a first-generation backcross to B6. The autosomal PWD content did not differ among the three *Msh2* genotype groups (Kruskal–Wallis *p* = 0.91), confirming that the observed fertility differences are not attributable to unequal genomic backgrounds (Figure 4A). A weak negative correlation between total autosomal PWD content and sperm count was detected (Spearman ρ = −0.42, *p* = 0.022, *n* = 29), consistent with the established relationship between heterosubspecific autosomal heterozygosity and spermatogenic impairment [7,8] (Figure 4B). This correlation did not remain significant after correction for multiple testing across all chromosomes. On the X chromosome, all 29 males carried the PWD allele at the *Hstx2* locus, confirming the microsatellite-based genotype assignment. The proximal region of Chr X, encompassing the recently described *Hstx3* locus [8], segregated between PWD and B6 alleles as a consequence of maternal recombination. The segregation of *Hstx3* and *Msh2* alleles did not deviate significantly from expectation (χ^2^ test, *p* = 0.14) (Figure 4C). Males carrying the B6 allele at Hstx3 showed nominally higher sperm counts (Figure 4D). The genome-wide scan of individual autosomal segments did not reveal any locus significantly associated with fertility after applying the Benjamini–Hochberg correction.

### 3.4. The Prdm9-Hstx2/Mir465 Incompatibility Overrides Msh2 Deficiency in F1 Hybrids

**Figure 5:**
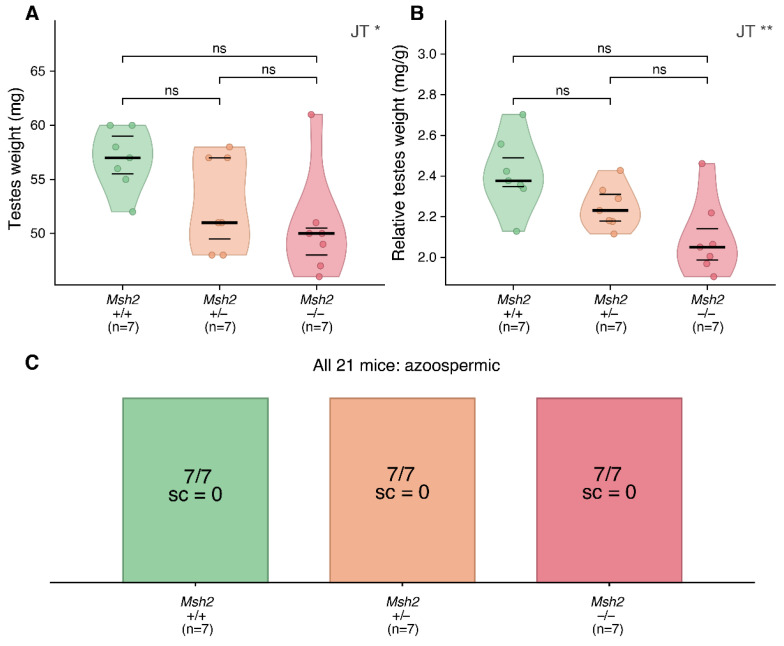
***Msh2* ablation does not rescue spermatogenic failure in (PWD × B6) F_1_ hybrid males.** Reproductive parameters of 21 (PWD × B6) F1 males segregating for *Msh2* (*n* = 7 *Msh2*^+/+^, 7 *Msh2*^+/−^, 7 *Msh2*^−/−^). (**A**) Testes weight (mg). Individual data points are shown as a jittered strip plot overlaid on a violin and box-and-whisker plot (*Msh2*^+/+^: green; *Msh2*^+/−^: orange; *Msh2*^−/−^: pink/red). Violins show kernel density; thick bar = median; thin bars = Q25/Q75. Pairwise comparisons by two-sided Mann–Whitney *U* test with BH-FDR correction across six F1 tests (testes weight + relative testes weight × three *Msh2* pairs). The “JT” annotation reports the Jonckheere–Terpstra trend test (single test per endpoint, BH within F1). All three pairwise contrasts ns; the JT trend is significant (*) (medians 57 vs. 50 mg). (**B**) Relative testes weight (mg g^−1^). All three pairwise contrasts ns; the JT trend is significant (**) (medians 2.38 vs. 2.05 mg g^−1^). Plot layout and statistical notation as in (**A**). (**C**) Spermatogenesis status: 7/7 F1 males in every *Msh2* group were azoospermic (sperm count = 0); sperm count was excluded from inferential testing. Significance thresholds: * *p* < 0.05, ** *p* < 0.01; ns, not significant.

While the BC1 design provided an initial test in a mixed genomic background, the canonical F1 hybrid cross represents the condition of full hybrid incompatibility. To evaluate whether *Msh2* deficiency can overcome the spermatogenic block under these conditions, we crossed congenic or near-congenic PWD-*Msh2*^−/−^ animals (N6 to N10) with B6 partners to generate F1 males directly. The (PWD × B6)*Msh*2^−/−^ F1 males showed slightly reduced testes weight compared to wild-type controls (50.6 ± 4.9 vs. 56.9 ± 2.9 mg, BH-adjusted *p* = 0.088), and no mature spermatozoa were recovered from any knockout male. Relative testes weight decreased monotonically across the *Msh2* genotype series (Jonckheere–Terpstra one-sided trend test, BH-adjusted *p* = 0.004; median rTW: wt 2.38%, KO/wt 2.23%, KO/KO 2.05%, *n* = 7 per group), mirroring the intrinsic detrimental effect of *Msh2* loss documented on the B6 background rather than providing any rescue (Figure 5A–C).

## 4. Discussion

Genetic dissection of *Prdm9*-driven hybrid male sterility in the house mouse has converged on a three-component model. According to this model, sterility requires the interaction of the *Prdm9* gene on chromosome 17 with the X-linked *Hstx2*/*Mir465* locus and *M. m. musculus*/*M. m. domesticus* autosomal heterozygosity [9,32]. Beyond these locus-specific incompatibilities, conserved meiotic surveillance mechanisms could in principle modulate this arrest. MSH2-mediated anti-recombination has been considered a candidate modifier. MSH2-guided mismatch recognition at meiotic recombination intermediates can destabilize heteroduplex DNA formed during strand invasion [20], and in budding yeast interspecific hybrids, meiotic repression of MSH2 alone was sufficient to restore spore viability [15]. Here, we asked whether this anti-recombination activity contributes to *Prdm9*-driven hybrid male sterility by combining a *Msh2* null allele with the (PWD × B6) F1 hybrid sterility model.

We have shown that complete loss of MSH2 function does not rescue spermatogenesis in (PWD × B6)F_1_ hybrid males, which remained azoospermic regardless of *Msh2* genotype. However, in BC1 males derived from the same cross and carrying the *Prdm9*^B6/PWD^–*Hstx2*^PWD^ allelic combination (PB/P), *Msh2* ablation produced a modest but significant partial rescue. *Msh2*^−/−^ PB/P males showed higher sperm counts and increased relative testes weight than their wild-type littermates, and the proportion of azoospermic individuals decreased from 38.5% in wild-type to 5.6% in *Msh2*^KO/KO^. The rescue was strictly recessive. Heterozygous animals were indistinguishable from wild-type controls. The effect was also *Hstx2/Mir465*-specific. No improvement was detected in BC1 males carrying the *Hstx2^B6^* allele, and on the fertile *Prdm9^B6/B6^* background, *Msh2* loss was instead associated with reduced sperm output, paralleling the modest negative effect of MSH2 deficiency observed on the pure B6 background.

The discrepancy between the F1 and BC1 results is informative and could be explained by postulating the superposition of two competing effects. The deleterious negative effect of MSH2 loss on spermatogenesis is observed in all genomic contexts. At the same time, in the hybrid background, MSH2-mediated mismatch recognition contributes an additional suppressive force on recombination at subspecifically diverged hotspots, and its removal partially relieves this suppression. In the BC1, where the overall incompatibility is attenuated by the reduced PWD genomic content, the benefit of relieving mismatch-dependent suppression outweighs the intrinsic cost of *Msh2* loss, resulting in a net improvement. In the F1, however, the *Prdm9*-*Hstx2*/*Mir465* incompatibility is at its maximum, and the intrinsic cost dominates. No rescue is possible because the primary block is too severe to be overcome by the removal of MMR alone. Together, these results position MSH2 as a modulatory rather than primary incompatibility factor, one whose contribution is genetically background-dependent and becomes detectable only when the dominant (Figure 5; Statistics S1) *Prdm9*-*Hstx2*/*Mir465* barrier is partially attenuated by genomic dilution.

The complete failure of *Msh2* ablation to restore fertility in F1 hybrids contrasts with the dramatic rescue achieved in *S. cerevisiae* × *S. paradoxus* hybrids, where meiotic repression of MSH2 increases spore viability up to 70-fold [15]. In yeast interspecific hybrids, the proximate cause of meiotic failure is mismatch-driven chromosome mis-segregation, making MSH2 loss directly restorative [13,14]. In the (PWD × B6) F1 hybrid, the primary barrier operates upstream of mismatch surveillance, through the asymmetric binding of PRDM9 to subspecifically diverged recombination hotspots and the activation of the *Mir465*-dependent pachytene checkpoint [7,9,24]. MSH2 loss does not resolve this asymmetry and does not alter the *Mir465* copy number that activates the checkpoint. The two upstream barriers are likely to account for the persistent sterility of F1 hybrids regardless of MMR status.

The modest rescue observed in BC1 males carrying the PB/P combination is consistent with MSH2 acting as a quantitative modifier of recombination intermediates downstream of these primary barriers. Mismatch recognition by MSH2 at heteroduplex DNA formed during strand invasion may destabilize a fraction of stalled intermediates and prevent their interhomolog repair [20]. Removing MSH2 could permit a proportion of these intermediates to complete repair, improving spermatogenic output without resolving the global asymmetry of PRDM9-directed DSBs. The strict recessivity indicates that one functional copy of *Msh2* is sufficient to saturate this activity. In mouse intrasubspecific hybrids with local nonhomology, MSH2 loss does not alter crossover frequencies but produces heteroduplex DNA retention in recombination products, suggesting that the meiotic threshold for heteroduplex rejection is higher than the mitotic threshold, possibly reflecting the mismatch tolerance of the meiosis-specific recombinase DMC1 [21]. A similar context dependence has been documented in *A. thaliana*, where *msh2* mutations rescue fertility only when the Class I crossover pathway is additionally blocked by *fancm zip4* mutations [18,19]. At the cytological level, autosomal asynapsis assessed by HORMAD2 staining decreased across the *Msh2* allelic series in BC1 hybrid males, with a significant one-sided trend but no significant pairwise differences.

The genotype-specific nature of the rescue places MSH2 within the genetic architecture of *Prdm9*-driven hybrid sterility. We previously demonstrated that two heterosubspecific autosomal pairs (Chr 17 and Chr 18) within an otherwise *M.m. domesticus* genome partially activate the *Prdm9*–*Hstx2* incompatibility, with a third pair (Chr 19) further enhancing the impairment [8]. In the BC1 males analyzed here, the sterile allelic combination was fixed by design, while the remaining autosomes segregated randomly. *Msh2* maps to chromosome 17 but is approximately 72 Mb from *Prdm9*, ensuring that the two loci segregate independently. MiniMUGA genotyping confirmed three to eleven PWD/B6 nonrecombinant chromosomes in BC1 males, consistent with the additive chromosomal model. The BC1 background therefore provides a range of incompatibility within which the modulatory effect of MSH2 becomes detectable. The preselection for *Hstx2*^PWD^ also introduced segregation at the linked *Hstx3* locus, and recombinant X chromosomes carrying B6 at *Hstx3* and PWD at *Hstx2* were associated with nominally higher sperm counts, consistent with the attenuating effect of *Hstx3*^B6^ [8].

It is tempting to speculate that the limited magnitude of the MSH2 effect even in this attenuated background reflects a reciprocal antagonism between the mismatch-surveillance machinery and the recombination proteins it monitors. Recent research in intraspecies budding yeast hybrids has shown that recombination proteins, including Rad51, Rad54, components of the ZMM complex, and the meiosis-specific recombinase DMC1, antagonize MSH2-containing complexes during meiosis [16,17]. If a comparable antagonism operates in mouse hybrid spermatocytes, the effective anti-recombination activity of MSH2 would already be partially buffered by the recombination machinery itself, providing one possible reason for its modest net effect. Direct assessment of the interplay between DMC1, ZMM proteins and MSH2 in hybrid spermatocytes would help to test this scenario.

Several limitations of the present study merit consideration. The cytological analyses of asynapsis, DMC1 foci and MLH3 crossovers were performed on a subset of BC1 males of the PB/P genotype, and extension to additional genotype combinations could refine the mechanistic model. The near-congenic status of the PWD *Msh2* line (N6–N10) introduces a residual B6 contribution, although the absence of rescue across all F1 males argues against this as a significant confound. Whether simultaneous removal of MSH2 and the SGS1 ortholog BLM would yield a stronger phenotype remains an open and experimentally testable question [15].

In conclusion, the current evidence is consistent with MSH2-mediated anti-recombination acting as a secondary modifier of *Prdm9*-driven hybrid male sterility rather than as a primary driver. It is tempting to speculate that mismatch-driven destabilization of recombination intermediates contributes to hybrid male sterility in mammals to a degree that is buffered by the meiotic recombination machinery itself.

## Data Availability

The data presented in this study are available within the article and its Supplementary Materials. The raw genotyping, cytology, and fertility datasets are available from the corresponding author upon reasonable request.

## Supplementary Materials

The following supporting information can be downloaded at: https://www.mdpi.com/article/10.3390/genes17070795/s1. Methods S1: Detailed protocols for CRISPR/Cas9 generation of the *Msh2*-knockout line, routine genotyping and cycling programmes, Sanger sequencing, the five-step MiniMUGA marker quality filter, and the statistical analysis framework. Table S1: Genotyping primers used in this study, with sequences, annealing temperatures, expected amplicon sizes, and references [4,6,33]. Table S2: Primary and secondary antibodies used for immunofluorescence and Western blotting, with clones, suppliers, and working dilutions. Table S3: Per-mouse fertility parameters (body weight, testes weight, sperm count, and relative testes weight) for all 250 males of the three cohorts (B6-*Msh2*, BC1, F1). Table S4: Per-cell cytology data (HORMAD2 asynapsis, DMC1 foci, MLH3 foci) for the B6-*Msh2* control cohort on the pure C57BL/6J background (one male per *Msh2* genotype). Table S5: Per-mouse and per-cell cytology data (HORMAD2, DMC1, MLH3) for the BC1 PB/P cohort, including the linear mixed-model results. Table S6: MiniMUGA SNP genotyping data, the five-step marker quality filter trace, per-chromosome genomic composition, and the associated confounding-locus statistics. Statistics S1: Complete inferential statistics workbook (descriptive summaries and all hypothesis tests for the B6-*Msh2*, BC1, F1, HORMAD2, DMC1 and MLH3 analyses). Figure S1: Cytological analysis of B6-*Msh2* males on a pure C57BL/6J background (asynapsis by HORMAD2/SYCP3, DMC1 foci across prophase I substages, and MLH3 crossover counts; *n* = 1 per *Msh2* genotype). Figure S2: Autosomal asynapsis frequency across the *Msh2* allelic series in BC1 PB/P males and its correlation with spermatogenic output (HORMAD2/SYCP3). Figure S3: DMC1 foci, MLH3 foci, and meiotic progression in BC1 PB/P males across *Msh2* genotypes, analyzed by linear mixed models. Figure S4: Genome-wide validation of subspecific genomic composition in PB/P BC1 males by MiniMUGA SNP genotyping (genome painting of controls and BC1 males and summary of autosomal composition). Figure S5: Representative spermatocyte nuclei illustrating immunofluorescence staining across consecutive stages of meiotic prophase I. (Each panel shows a representative cell of a given prophase I substage, labelled with the antibodies and markers indicated in the corresponding panel.)

## Abbreviations

The following abbreviations are used in this manuscript:

B/B, B/P, PB/B, PB/P	*Hstx2*/*Prdm9* allelic-combination shorthand (B = B6 allele; P = PWD allele)
B6	C57BL/6J inbred strain
BC1	first backcross generation
BCA	bicinchoninic acid assay
BH	Benjamini–Hochberg
BSA	bovine serum albumin
CCD	charge-coupled device
CCP	Czech Centre for Phenogenomics
CRISPR/Cas9	clustered regularly interspaced short palindromic repeats/CRISPR-associated protein 9
DAPI	4′,6-diamidino-2-phenylindole
DSB	DNA double-strand break
ECL	enhanced chemiluminescence
F1	first filial generation
FDR	false discovery rate
HRP	horseradish peroxidase
IMG CAS	Institute of Molecular Genetics of the Czech Academy of Sciences
IQR	interquartile range
JT	Jonckheere–Terpstra
KO	knockout
LMM	linear mixed model
MiniMUGA	Miniature Mouse Universal Genotyping Array
MMR	DNA mismatch repair
OR	odds ratio
PBS	phosphate-buffered saline
PCR	polymerase chain reaction
PVDF	polyvinylidene difluoride
PWD	PWD/Ph inbred strain
RIPA	radioimmunoprecipitation assay buffer
rTW	relative testes weight
SC	sperm count
SDS-PAGE	sodium dodecyl sulphate–polyacrylamide gel electrophoresis
sgRNA	single-guide RNA
SNP	single-nucleotide polymorphism
SPF	specific pathogen-free
SSLP	simple sequence length polymorphism
wt	wild type
