# Supplementary material for: The Role of the Msh2 Mismatch Repair Gene in the Prdm9-Driven Hybrid Male Sterility in the House Mouse
Source: Genes (Basel). 2026 Jul 12;17(7):795. doi: 10.3390/genes17070795 (PMC13409646; doi:10.3390/genes17070795)
Supplement: Supplementary file 1 [file genes-17-00795-s001.zip › figureS1-S5.pdf]

Supplementary figures

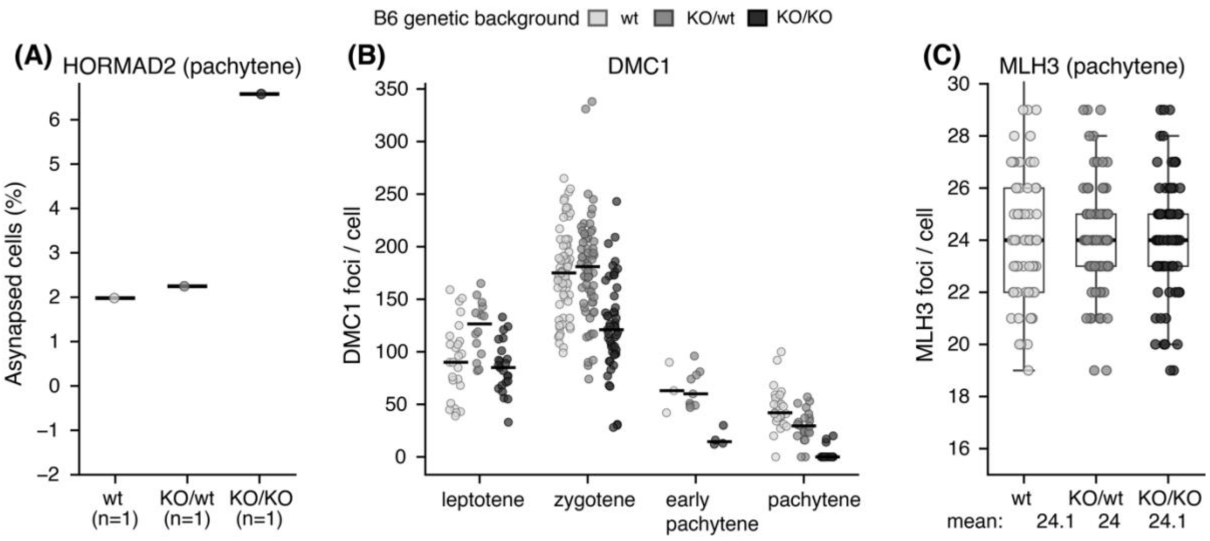

Supplementary Figure S1: Cytological analysis of B6-*Msh2* males on a pure C57BL/6J background. One B6-*Msh2* male of each *Msh2* genotype on a pure C57BL/6J background was analysed to determine whether *Msh2* genotype affects meiotic markers in a non-hybrid context: C2354 (*Msh2*$^{+/+}$), C2352 (*Msh2*$^{+/-}$) and C2353 (*Msh2*$^{-/-}$). With $n = 1$ male per genotype, no inferential statistics are applied; per-cell raw counts are provided in Table S6. **(A)** Percentage of pachytene spermatocytes carrying at least one asynapsed autosome (HORMAD2/SYCP3 co-staining). Asynapsis is uniformly low (≤ 7%), as expected for a non-hybrid B6 background. **(B)** Total DMC1 foci per spermatocyte across prophase I substages. Each dot is one nucleus; horizontal bars indicate per-mouse medians. **(C)** MLH3 foci per pachytene spermatocyte (mean foci per cell indicated below each column).

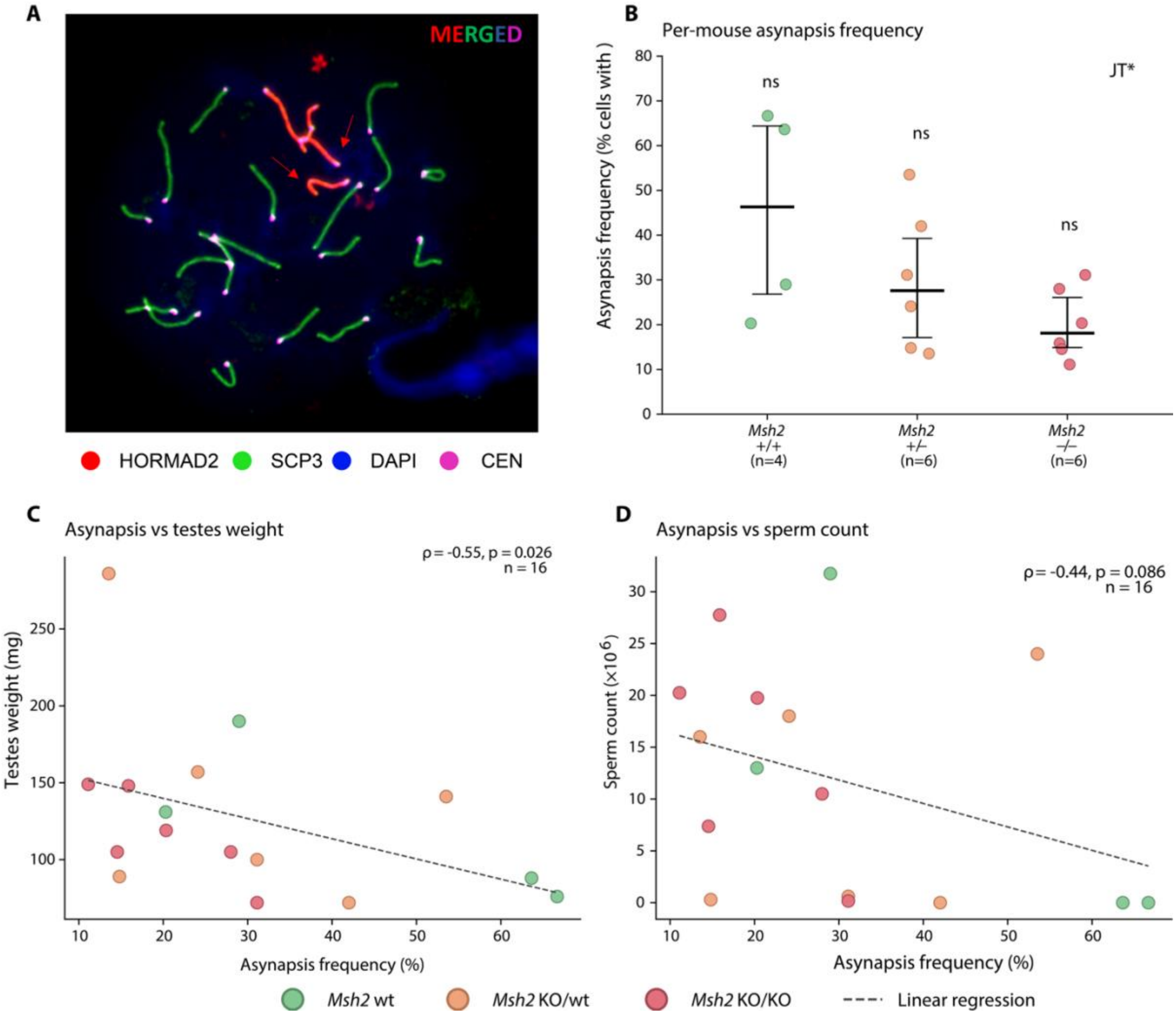

Supplementary Figure S2. Autosomal asynapsis frequency in BC1 PB/P males decreases with *Msh2* copy number and correlates with spermatogenic output. (A) An asynapsed primary spermatocyte in prophase I of the pachytene stage, stained by immunofluorescence **(B)** Per-mouse frequency of pachytene spermatocytes carrying at least one HORMAD2-positive asynapsed autosome according to *Msh2* genotype in the BC1 PB/P cohort (*Msh2*$^{+/+}$ n = 4, *Msh2*$^{+/-}$ n = 6, *Msh2*$^{-/-}$ n = 6). Horizontal bars represent the median and interquartile range (IQR). **(C)** Relationship between per-mouse asynapsis frequency and testis weight. The dashed line indicates the linear regression fit (Spearman ρ = −0.55, n = 16). **(D)** Relationship between per-mouse asynapsis frequency and sperm count. The dashed line indicates the linear regression fit (Spearman ρ = −0.44, n = 16). Statistical significance is indicated as follows: * $p < 0.05$, ** $p < 0.01$, *** $p < 0.001$, ns $p \geq 0.05$.

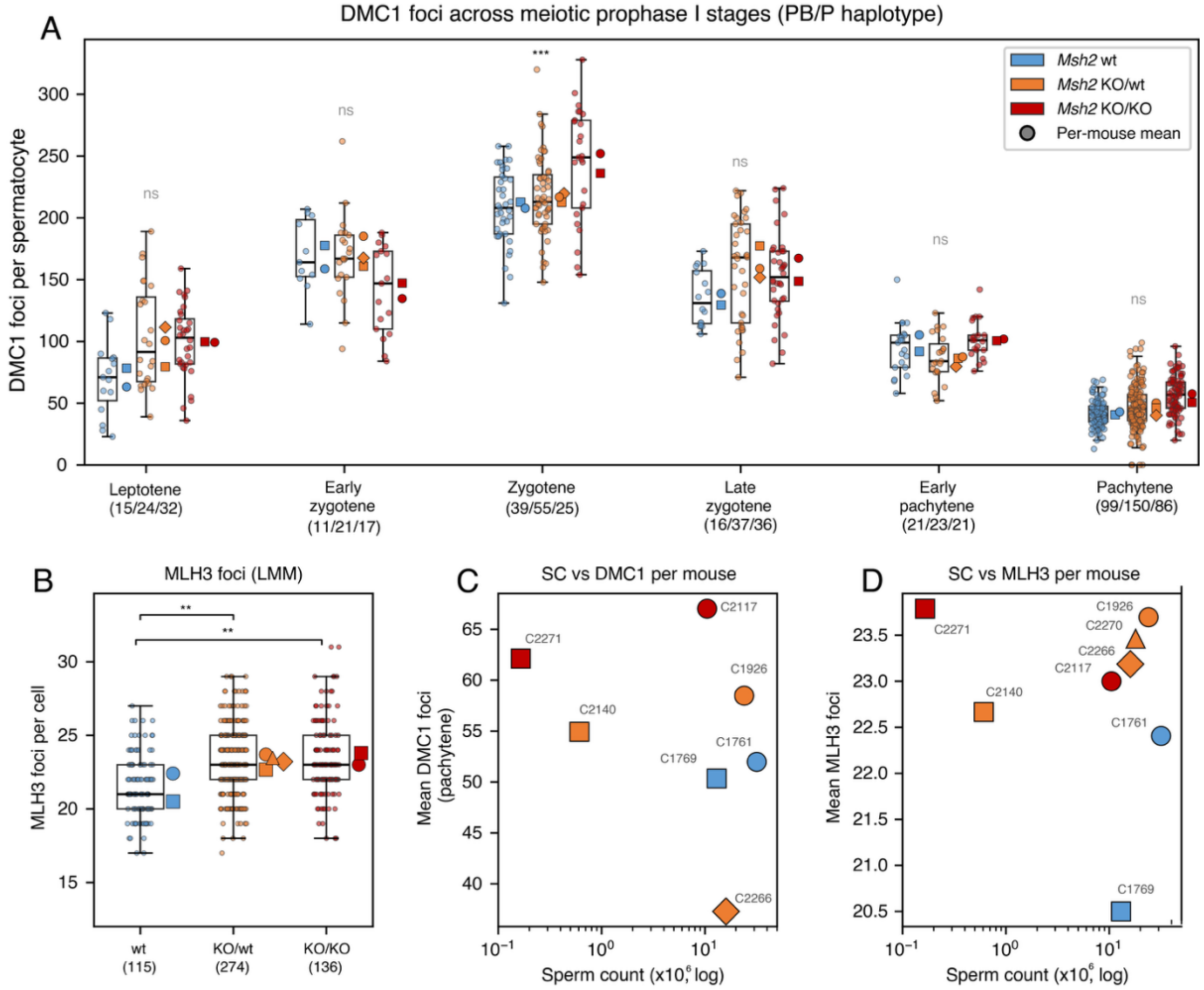

Supplementary Figure S3: DMC1 foci, MLH3 foci, and meiotic progression in PB/P BC1 males across *Msh2* genotypes. All analyses are restricted to the PB/P allelic combination (*Prdm9*$^{B6/PWD}$, *Hstx2*^{PWD}) of BC1 backcross males. Small dots are individual spermatocytes. Large symbols are per-mouse means, with a unique symbol shape per mouse within each genotype. Horizontal bars indicate the per-cell median. **(A)** Total DMC1 foci (autosomal + XY) per spermatocyte across meiotic prophase I stages. Significance was assessed by linear mixed models (LMM) with Msh2 genotype as fixed effect and mouse identity as random intercept. Cell counts per genotype are indicated in parentheses (wt / KO/wt / KO/KO). Msh2 wt, n = 2 mice; KO/wt, n = 3; KO/KO, n = 2. **(B)** MLH3 foci per spermatocyte at pachytene (LMM). Msh2 wt, n = 2 mice (115 cells); KO/wt, n = 4 (274 cells); KO/KO, n = 2 (136 cells). **(C)** Relationship between sperm count and mean DMC1 foci (pachytene) per individual mouse. **(D)** Relationship between sperm count and mean MLH3 foci per individual mouse. In panels C and D, each point represents one mouse. *p < 0.05; **p < 0.01; ***p < 0.001; ns, not significant.

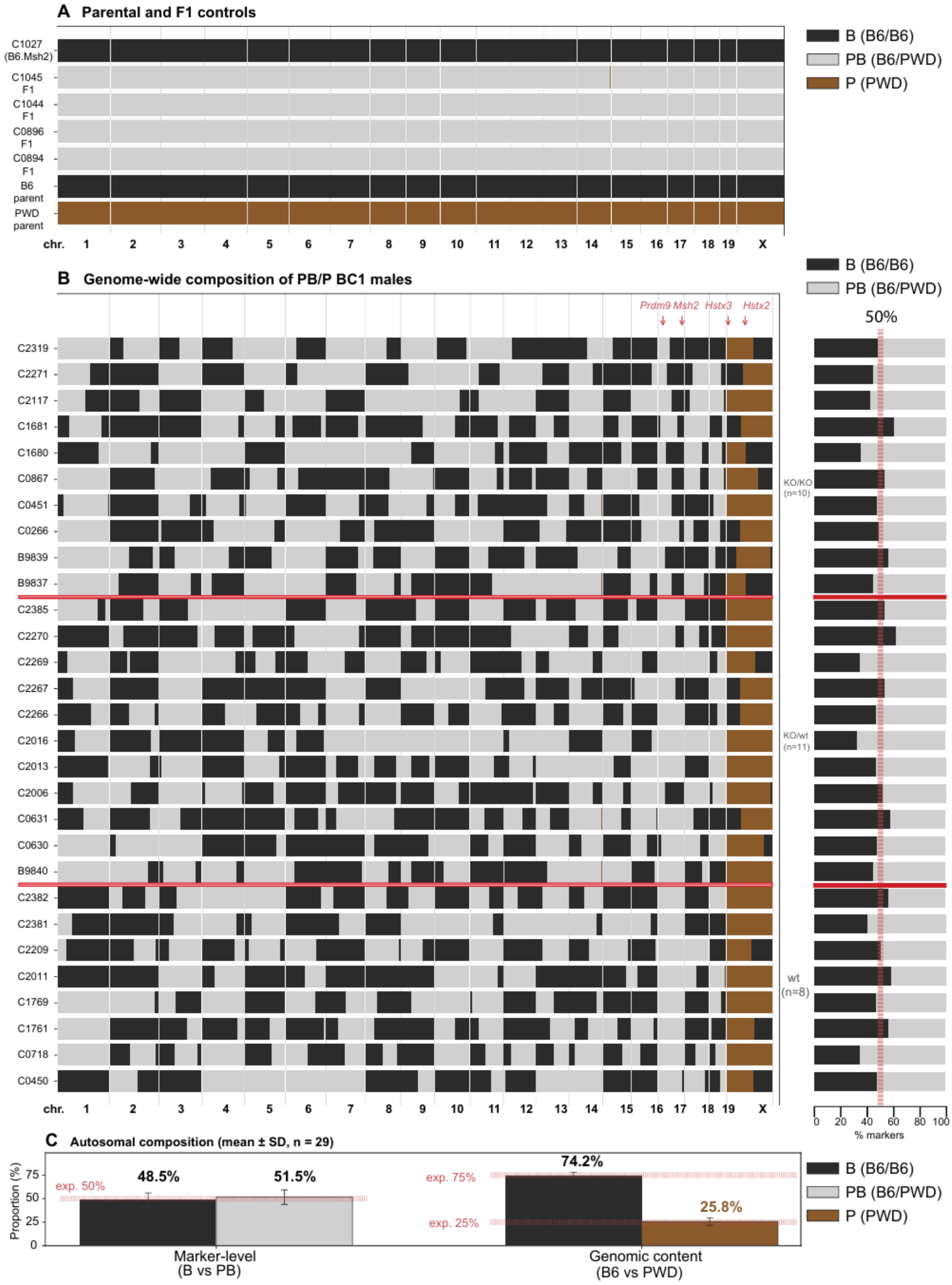

Supplementary Figure S4. Genome-wide validation of subspecific genomic composition in PB/P BC1 males by MiniMUGA SNP genotyping. (**A**) Genome painting of parental and F1 controls. Each horizontal track represents one individual with I.D.; each vertical segment corresponds to chromosome 1 -19 and X, coloured by subspecific genotype: B6/B6 homozygous (B, dark grey), B6/PWD heterozygous (PB, light grey), or PWD hemizygous on the X chromosome (P, brown). The PWD parental strain, B6 parental strain, four F1 hybrid females (IDs: C0894, C0896, C1044, C1045),

and the B6-*Msh2* male (C1027) are shown. A total of 346 markers producing discordant or unreliable genotype calls were identified through a five-step quality filter and excluded from all subsequent analyses, leaving 3,270 informative markers. (**B**) Genome-wide composition of 29 PB/P BC1 males, ordered by *Msh2* genotype (wt, KO/wt, KO/KO). Autosomes (1–19) display the expected BC1 segregation pattern with either homozygous B6/B6 or heterozygous B6/PWD segments, including recombinant chromosomes from maternal crossovers. On Chr X (hemizygous), males carry either the full PWD genotype or a recombinant X with a B6 proximal segment and PWD distal segment. Red arrows indicate the positions of *Prdm9* (~15.6 Mb) and *Msh2* (~87.7 Mb) on Chr 17, and the *Hstx3* (0–7.2 Mb) and *Hstx2* (66.5–69.2 Mb) loci on Chr X (all coordinates GRCm39). Horizontal stacked bars to the right show the proportion of B (dark) versus PB (light) autosomal markers per individual; the dashed red line indicates the expected 50%. (**C**) Summary of autosomal genomic composition across all 29 BC1 males (mean ± SD). Left: marker-level proportions (B = 48.5 ± 7.6%, PB = 51.5 ± 7.6%), consistent with the expected 50:50 segregation. Right: estimated actual genomic content, calculated as B6% = %B + 0.5 × %PB and PWD% = 0.5 × %PB (B6 = 74.2 ± 3.8%, PWD = 25.8 ± 3.8%), closely matching the expected 75:25 ratio for a first-generation backcross.

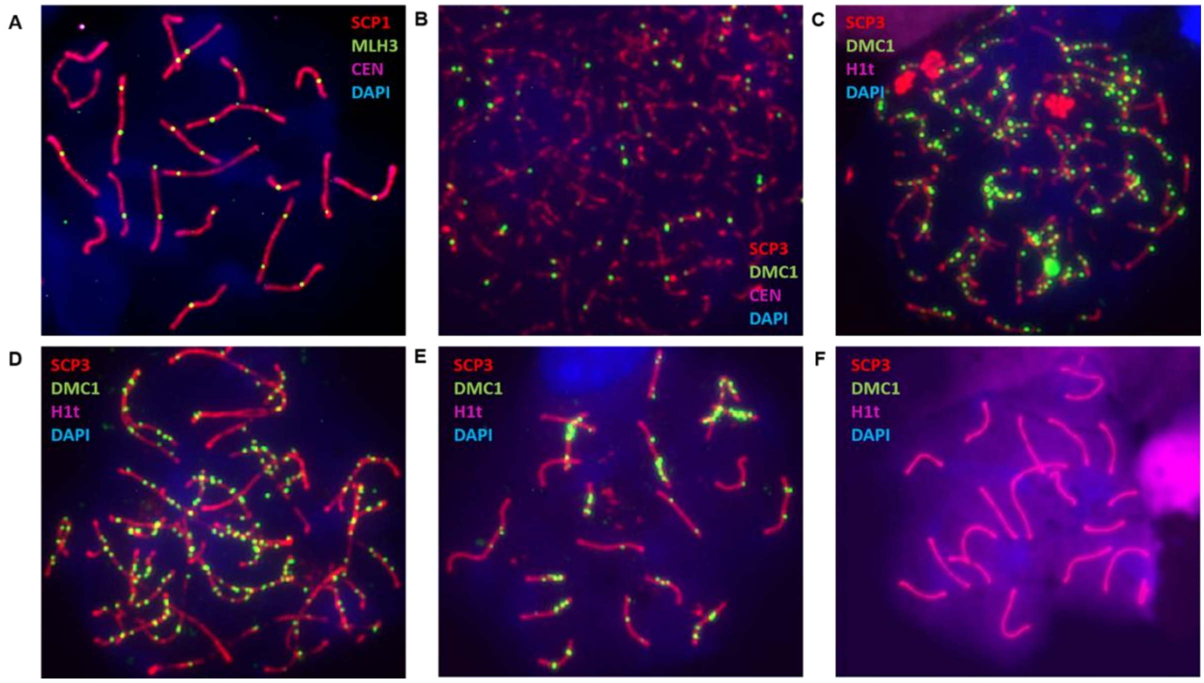

Supplementary Figure S5. Representative spermatocyte nuclei illustrating immunofluorescence staining across consecutive stages of meiotic prophase I. Each panel shows a representative cell of a given prophase I substage, labelled with the antibodies against markers indicated in the corresponding panel. **(A)** Mid-pachytene nucleus stained for SYCP1 (red), MLH3 (green), centromeres (CEN, magenta) and DNA (DAPI, blue). (**B–F**) Nuclei stained for SYCP3 (red), DMC1 (green), H1t (magenta) and the DNA (DAPI - blue). **(B)** Leptotene. **(C)** Zygotene. **(D)** Late zygotene. **(E)** Early pachytene. **(F)** Late pachytene, in which an H1t-positive signal is already detectable
