# Supplementary material for: The Role of the Msh2 Mismatch Repair Gene in the Prdm9-Driven Hybrid Male Sterility in the House Mouse
Source: Genes (Basel). 2026 Jul 12;17(7):795. doi: 10.3390/genes17070795 (PMC13409646; doi:10.3390/genes17070795)
Supplement: Supplementary file 1 [file genes-17-00795-s001.zip › Methods_S1.pdf]

Supplementary Methods S1

Detailed protocols for mouse line generation, genotyping, Sanger sequencing, MiniMUGA analysis, and statistical methods

Companion file to Fusek et al., 'The role of mismatch repair gene Msh2 in Prdm9-driven hybrid male sterility in the house mouse model'

Contents

S1. Generation of the Msh2 null mutant line and genotyping

S1.1 Generation of the Msh2 null mutant line (CRISPR/Cas9)

S1.2 DNA extraction

S1.3 PCR setup and master mixes

S1.4 Cycling programs

S1.5 Agarose gel electrophoresis

S1.6 Sanger sequencing

S2. MiniMUGA genotyping and quality filtering

S2.1 Sample submission and array platform

S2.2 Five-step marker quality filter

S2.3 Downstream interpretation

S3. Statistical analysis

S3.1 Software and reproducibility

S3.2 Descriptive statistics

S3.3 Inferential framework (fertility cohorts)

S3.4 Multiple-testing correction

S3.5 Cytology analyses: per-mouse vs per-cell scoring and choice of test

S3.6 Linear mixed models for DMC1 and MLH3

S1. Generation of the *Msh2* null mutant line and genotyping

S1.1 Generation of the *Msh2* null mutant line

The *Msh2* knockout line was generated on the C57BL/6J (B6) background in collaboration with the Transgenic Unit of the Czech Centre for Phenogenomics (CCP), Institute of Molecular Genetics (IMG) of the Czech Academy of Sciences, using CRISPR/Cas9 genome editing. Guide RNAs (sgRNAs) were designed using CHOPCHOPv2 and CCTop with the canonical

5'-NGG-3' protospacer adjacent motif (PAM) sequence for *Streptococcus pyogenes* Cas9 (SpCas9). Three sgRNAs targeting exon 7 of *Msh2* were used in combination; their target protospacer sequences are listed below.

sgRNA	Target protospacer (5'→3')	Locus
sgRNA-1	TAACCGGCTTGCCAAGAAAT	Msh2 exon 7
sgRNA-2	GAGACAAGCAGCGAATTTAC	Msh2 exon 7
sgRNA-3	AGACTGTTACCGACTGTATC	Msh2 exon 7

B6 females (8–10 weeks) were superovulated and mated with B6 males. Fertilised zygotes were collected from oviducts. Cas9 ribonucleoprotein (RNP) complexes pre-assembled with the three sgRNAs were introduced into zygotes by electroporation, and embryos were transferred into pseudopregnant surrogate females. Founder animals were identified by PCR amplification of the targeted region, resolved by polyacrylamide gel electrophoresis (PAGE) and, performed by the Transgenic Unit of the Czech Centre for Phenogenomics (CCP) confirmed by Sanger sequencing (Section S1.6). The selected founder carried the deletion in mosaic form. To resolve the germline mosaicism, founder progeny were backcrossed to wild-type B6 mice for two generations, after which the deletion allele was established and the 29 bp deletion was re-confirmed by Sanger sequencing. The deletion allele removes a 29 bp segment within exon 7 of Msh2 (chr17:87,996,444-87,996,472; GRCm39/mm39; Figure 1A). The deletion induces a frameshift predicted to cause premature translation termination downstream of the editing site. Loss of full-length MSH2 protein in homozygous animals was confirmed by Western blotting (see main text, Materials and Methods). The line was maintained in the heterozygous state, and homozygous animals as well as the heterozygous knockout carriers used in the experimental crosses were derived from this heterozygous line. To prevent genetic drift and any potential influence on the genetic background, the line was backcrossed to wild-type B6 mice every three generations. Direct genome editing in PWD/Ph embryos was attempted using an *in vivo* oviduct electroporation approach (the GONAD method), but successful germline transmission was not achieved in this strain. The *Msh2* null allele was therefore transferred onto the PWD background by serial backcrossing using fertile heterozygous female carriers (see main text, Materials and Methods).

S1.2 DNA extraction

Crude NaOH lysis (routine genotyping)

For routine genotyping, tail-tip biopsies (~2 mm) were lysed in 75 µL of 50 mM NaOH at 95 °C for 1.5 h, neutralised by addition of 7.5 µL of 1 M Tris-HCl pH 8.0, briefly vortexed, centrifuged at 14 000 × g for 1 min, and stored at −20 °C until use. One microlitre of crude lysate was used per PCR reaction.

Column-based purification (for Sanger sequencing)

For Sanger sequencing, partially purified DNA was isolated from tail-tip or spleen tissue (~5 mg) using the Puregene Core Kit A (Qiagen, cat. no. 158267) according to the manufacturer's instructions. DNA concentration and purity (A260/A280 ratio of 1.8–2.0) were assessed on a

NanoDrop One spectrophotometer (Thermo Fisher Scientific), and samples were adjusted to 30 ng/µL in 10 mM Tris-HCl pH 8.0.

S1.3 PCR setup and master mixes

Master mix for Programs A and B (DreamTaq)

Standard genotyping reactions were assembled in 15 µL of master mix combined with 1 µL of DNA template (16 µL total volume):

Component	Stock	Volume (µL) per 16 µL rxn	Final concentration
Nuclease-free water	—	11.95	—
dNTPs	10 mM each	0.30	0.2 mM each
Primers F + R (mixed)	10 µM each	0.375	0.25 µM each
10× DreamTaq Buffer + 20 mM $MgCl_2$	10×	1.50	1× / 2 mM $MgCl_2$
DreamTaq DNA Polymerase (Thermo EP0701)	5 U/µL	0.12	0.6 U / rxn
DNA template (crude NaOH lysate)	—	1.00	—
**Total**		**15 + 1 (DNA) = 16**	

Master mix for Program C (Prdm9 Exon 12 sequencing)

Following the published protocol of Mukaj et al. (2020) [1], each reaction contained 50–100 ng of spleen-derived DNA (Puregene Core Kit A, Qiagen 158267), 0.4 µM of each primer (Exon12-L1 and Exon12-R), 0.2 mM dNTPs, 2 mM $MgCl_2$, and 0.15 U of Taq DNA Polymerase, recombinant (Thermo Scientific, cat. no. EP0404).

S1.4 Cycling programs

All amplifications were performed on a Bio-Rad T100 Thermal Cycler. Three cycling programs were used in this study.

Program A — *Msh2* standard

Default program for *Msh2* wild-type and knockout allele genotyping.

Step	Temperature	Time	Cycles
Initial denaturation	95 °C	3 min	1
Denaturation	95 °C	30 s	30
Annealing	60 °C	30 s	30
Extension	72 °C	30 s	30
Final extension	72 °C	5 min	1

Hold	10 °C	∞	—

Program B — SSLP touchdown (also tolerated by *Msh2* primers)

Program for amplification of microsatellite markers (M334, SR51); also used on occasion for *Msh2* primer pairs with comparable performance to Program A.

Step	Temperature	Time	Cycles
Initial activation	95 °C	2 min	1
Denaturation	95 °C	15 s	5 (touchdown)
Annealing	68 → 58 °C (−2 °C/cycle)	15 s	5 (touchdown)
Extension	72 °C	15 s	5 (touchdown)
Denaturation	95 °C	15 s	25
Annealing	58 °C	15 s	25
Extension	72 °C	15 s	25
Final extension	72 °C	5 min	1

Program C — Prdm9 Exon 12 sequencing

Program for amplification and Sanger sequencing of *Prdm9* Exon 12 (zinc-finger array), used for verification of parental and F1 hybrid genotypes. Adapted from Mukaj et al. (2020) [1].

Step	Temperature	Time	Cycles
Initial denaturation	94 °C	5 min	1
Denaturation	94 °C	30 s	40
Annealing	61 °C	1 min	40
Extension	68 °C	2 min	40
Final extension	72 °C	7 min	1

S1.5 Agarose gel electrophoresis

PCR products were mixed with 6× DNA loading dye (Thermo Fisher Scientific, cat. no. R0611) and resolved on agarose gels in 0.5× TBE buffer at 100 V for 35–45 min. Gel percentages were optimised per locus: 2% for *Msh2* (sufficient to resolve the 29 bp size difference between wt and KO alleles), 4% for SSLP markers (M334, SR51), and 1.5% for *Prdm9* Exon 12 amplicons (~1.1 kb). Gels were stained with ethidium bromide (0.5 µg/mL) and visualised under UV illumination on a Bio-Rad Gel Doc imaging system. Amplicon sizes were estimated against a 100 bp DNA ladder (Thermo SM0241) or GeneRuler 1 kb Plus (Thermo SM1331), depending on expected product size. Wild-type and deletion controls were included on each gel. Ambiguous reactions were repeated from a fresh DNA preparation.

S1.6 Sanger sequencing

For high-confidence verification of selected genotypes, Sanger sequencing was performed. Amplicons were resolved on agarose gels as described above; bands of interest were excised under long-wavelength UV illumination (365 nm) to minimise UV-induced DNA damage, and purified using the MinElute Gel Extraction Kit (Qiagen, cat. no. 28606) following the manufacturer's protocol, with elution in 12 µL of Buffer EB. Purified amplicons were quantified on a NanoDrop One spectrophotometer and adjusted to 10–20 ng/µL depending on amplicon length. Sequencing reactions were submitted to the SeQme sequencing service (Dobříš, Czech Republic) and performed bidirectionally using the forward and reverse PCR primers at 25 pmol per reaction. Resulting chromatograms were inspected manually for base-calling quality, trimmed at both ends to remove low-quality bases (Phred Q < 20), and aligned to the C57BL/6J reference genome (GRCm39/mm39) using BioEdit (v7.2). Sequence identity was verified by NCBI BLAST against the *Mus musculus* reference assembly; variant calls were confirmed by visual inspection of overlapping forward and reverse chromatograms.

This workflow was applied to (i) verification of the CRISPR-induced 29 bp deletion in the *Msh2* exon 7 founder and subsequent generations, and (ii) verification of *Prdm9* alleles in parental strains, F1 hybrids, and B6-*Msh2* founders entering the BC1 cross (when only a limited number of breeding-cage parents required confirmation).

S2. MiniMUGA genotyping and quality filtering

S2.1 Sample submission and array platform

MiniMUGA genotyping and genomic background validation

A subset of 29 BC1 males carrying the sterile PB/P haplotype ($Prdm9^{B6/PWD}$, $Hstx2^{PWD}$) together with 5 parental controls (one B6-$Msh2^{+/-}$ male, and 4 F1 hybrid females) were genotyped using the MiniMUGA array (Neogen EUROPE, The Dairy School, South Ayrshire) MiniMUGA is a low-density SNP platform providing approximately 11,000 markers across the mouse genome, designed for strain verification, quality control, and subspecific origin assignment.

Genomic DNA was extracted from tail or spleen tissue as described above. Samples were processed by Neogen Corporation. Raw genotype calls were converted to subspecific origin (B6, PWD, or heterozygous B6/PWD) by comparison with reference genotypes from the parental PWD/Ph and C57BL/6J strains genotyped on the same platform. Markers were filtered in five steps: (i) markers producing discordant calls in the parental PWD or B6 strains, (ii) markers discordant in two or more F1 hybrid controls, (iii) markers discordant in the B6-Msh2 control (C1027), (iv) heterozygous (PB) calls on the hemizygous X chromosome in males, and (v) isolated singleton genotype calls flanked by markers of a different genotype in BC1 individuals. This procedure removed 346 markers, leaving 3,270 informative subspecies-discriminating markers across autosomes (chromosomes 1–19), and the X chromosome. All coordinates are reported in the mm10 (GRCm38) reference assembly.

For each BC1 male, the subspecific genotype (B6/B6 or B6/PWD) was determined at each autosomal marker. The autosomal PB fraction was calculated as the proportion of heterozygous (PB) markers out of all called autosomal markers. For the hemizygous X

chromosome, PB calls (which are biologically impossible in males) were treated as artefacts and excluded. The allelic status at the *Hstx3* locus was determined from the most proximal informative X-linked marker (SXX200249422 at 6.24 Mb, mm10), located within the 0–7.23 Mb interval defined [9]. The *Hstx2* locus status was confirmed using three markers within the 66.5–69.2 Mb interval (mm10).

S2.2 Five-step marker quality filter

Raw MiniMUGA genotype calls were processed through a five-step marker quality filter designed to remove technical artifacts and platform-specific call errors before downstream analysis. The filter exploits the controlled genetic structure of the experimental cohort (defined parental strains, F1 controls of known heterozygosity, B6-Msh2 control males of defined background, and male-only X-chromosome hemizygosity) to flag and remove markers that produce calls inconsistent with these expectations. The five steps are applied sequentially and are summarised below.

Step	Filter name	Rationale	Comparator group used
1	Parental control discordance	Markers giving genotype calls in parental B6 or PWD samples inconsistent with the homozygous expectation for that strain were removed.	Parental B6 and PWD/Ph
2	F1 heterozygosity check	Markers failing to call heterozygous in B6xPWD)F1 control samples (where heterozygosity is genetically certain) were removed.	(B6xPWD)F1 control hybrids female
3	B6.*Msh2* background check	Markers showing non-B6 calls in B6.*Msh2* males (background should be ~100% B6 outside the Msh2 region) were removed.	B6.*Msh2* males
4	X-chromosome hemizygosity	X-chromosome markers failing to call hemizygous in male samples (no heterozygous calls expected) were removed.	All male samples
5	BC1 singleton check	Markers showing only a single non-B6 call across the entire BC1 cohort were flagged as likely artifacts and removed.	All BC1 cohort samples

Marker call counts per step depend on the exact sample composition of the genotyping run; the cumulative result for the present cohort is 346 markers removed, leaving 3 270 informative markers retained for downstream analysis. Per-marker filter trace and per-mouse genotype calls are provided in Table S6. Visual summaries (genome painting and analytical validation panels) are provided in Figures 5 and S4.

S2.3 Downstream interpretation

Filtered marker calls were used to (i) confirm that BC1 males carry approximately 75% B6 and 25% PWD autosomal genome on average, in agreement with theoretical expectation for a

one-generation backcross to B6; (ii) visualise per-chromosome distribution of subspecific segments in individual males by 'genome painting'; and (iii) verify the genomic context of *Msh2*, *Prdm9*, and *Hstx2* segregation across BC1 progeny.

S3. Statistical analysis

S3.1 Software and reproducibility

All statistical analyses were performed in Python (v3.11) using NumPy, pandas, the scipy.stats module (rank-based and exact tests), and statsmodels (linear mixed models). Source data for every reported test are provided in Tables S3 (fertility), S4 (B6-*Msh2* control cytology) and S5 (BC1 cytology). Complete inferential output, including descriptive statistics, omnibus tests, pairwise comparisons, trend tests, linear mixed models, and Benjamini–Hochberg-adjusted p-values for every endpoint is reported in Statistics S1 (multi-sheet).

S3.2 Descriptive statistics

Continuous endpoints (testis weight, relative testis weight, sperm count) are reported as median [interquartile range, IQR] in the main text. Means ± standard deviation and full ranges are additionally reported in the descriptive sheets of Statistics S1. Sperm count was $\log_{10}$-transformed for visualisation but inferential tests were performed on the original (untransformed) values using rank-based non-parametric statistics. Cytological endpoints are reported as per-mouse medians or, for binary autosomal asynapsis (HORMAD2), as the percentage of asynapsed pachytene cells per male.

S3.3 Inferential framework (fertility cohorts)

Normality of residuals within each group was assessed using the Shapiro–Wilk test. Because multiple groups violated the normality assumption, non-parametric tests were used throughout for fertility endpoints.

Mann–Whitney U test (MWU): two-sided pairwise comparisons between *Msh2* genotypes (wt, KO/wt, KO/KO) within each cohort or each haplotype subgroup of the BC1 cohort. Rank-biserial correlation reported as standardised effect size.

Kruskal–Wallis test (KW): omnibus comparison across the three *Msh2* genotypes within each subgroup. KW results are reported descriptively (without Benjamini–Hochberg correction) and serve as a global check supporting the pairwise MWU output.

Jonckheere–Terpstra trend test (JT): one-sided test for monotonic ordering of group medians across the Msh2 genotype gradient wt → KO/wt → KO/KO. Used as the primary test for monotonic dose-response patterns. The direction is fixed a priori, and one-sided p-values with z-statistics are reported.

Fisher's exact test: two-sided comparison of binary outcomes. Used for the azoospermia rate (sperm count = 0) within the BC1 PB/P haplotype.

S3.4 Multiple-testing correction

Multiple-testing correction was applied using the Benjamini–Hochberg false discovery rate procedure (BH-FDR), grouped by cohort and endpoint as follows:

B6 cohort: BH across 9 MWU tests (3 Msh2 pairs × 3 endpoints: testis weight, sperm count, relative testis weight).

BC1 cohort: BH across 8 JT trend tests (2 endpoints × 4 haplotypes) and across 24 MWU pairwise tests (3 pairs × 2 endpoints × 4 haplotypes). Testis-weight pairwise comparisons (12 tests) are reported descriptively only, because absolute testis weight was confounded by body-weight variation in the BC1 cohort; relative testis weight is used as the corrected secondary endpoint. The Fisher exact azoospermia test was not BH-corrected.

F1 cohort: BH across 6 MWU tests (3 pairs × 2 endpoints: testis weight, relative testis weight). Sperm count was excluded from inferential testing because all F1 males were azoospermic.

HORMAD2 cytology (BC1 PB/P): the Jonckheere–Terpstra one-sided trend test as the primary test, with BH-FDR across the 3 MWU pairwise tests (single endpoint: % asynapsed pachytene cells). LMM analyses of DMC1 and MLH3 (Section S3.6) report uncorrected p-values; given the small number of mice per Msh2 group, these tests should be interpreted as exploratory.

Significance thresholds: ns $p \geq 0.05$; * $p < 0.05$; ** $p < 0.01$; *** $p < 0.001$. Significance brackets in figures reflect the BH-adjusted p-value where BH was applied.

S3.5 Cytology analyses: per-mouse vs per-cell scoring and choice of test

Cytological data are inherently hierarchical: each male contributes multiple scored nuclei. The appropriate unit of analysis depends on the per-group sample size and the biological question. In this study, two complementary strategies are used:

(i) Per-mouse summaries with non-parametric tests (HORMAD2). Where the cohort is large enough to support per-mouse inferential testing (n = 4 wt, 6 KO/wt, 6 KO/KO for HORMAD2 in PB/P), the per-mouse proportion of asynapsed pachytene cells is the unit of analysis. This avoids pseudoreplication and matches the unit at which biological variability is most relevant (between mice rather than between cells within a mouse). Tests used are the Jonckheere–Terpstra one-sided trend test (primary), with KW omnibus and MWU pairwise as secondary (Section S3.3).

(ii) Linear mixed models with per-cell observations (DMC1, MLH3). For DMC1 (n = 2 wt, 3 KO/wt, 2 KO/KO) and MLH3 (n = 2 wt, 4 KO/wt, 2 KO/KO) in PB/P, the per-mouse sample size is too small to support reliable non-parametric per-mouse inference. To leverage the much larger number of per-cell observations (typically 50–200 cells per mouse) while accounting for the nested data structure, linear mixed models with mouse identity as a random intercept were used (Section S3.6). This approach follows Jasin lab convention for per-cell focus counts in mouse meiocytes [3]. p-values from these LMMs are reported uncorrected and are interpreted as exploratory given the limited number of mice.

The choice of primary test for each marker is summarised below:

Marker	Endpoint	Per-mouse n (wt / KO/wt / KO/KO)	Primary test	Reported in
HORMAD2	% asynapsed pachytene cells	4 / 6 / 6	KW omnibus, MWU pairwise, Spearman vs fertility (per-mouse unit)	Statistics S1: HORMAD2_* sheets
DMC1	Total foci per spermatocyte by substage	2 / 3 / 2	LMM (per-cell unit, mouse random intercept)	Statistics S1: DMC1_LMM_BC1, Descriptive_DMC1_BC1
MLH3	Pachytene foci per spermatocyte; bivalents without MLH3 focus	2 / 4 / 2	LMM (per-cell unit, mouse random intercept)	Statistics S1: MLH3_LMM_BC1

S3.6 Linear mixed models for DMC1 and MLH3

Linear mixed models (LMM) were fitted in statsmodels (mixedlm) with the structure:

response ~ Msh2_genotype + (1 | mouse_id)

where the response is the per-cell focus count, Msh2_genotype is a fixed effect (reference = wt), and mouse_id is a random intercept. The random intercept on mouse identity absorbs between-mouse heterogeneity in baseline focus counts and propagates it into the standard errors of the fixed-effect coefficients, so that inference is effectively at the mouse level rather than the cell level. Model coefficients are interpreted as the average change in per-cell focus count for KO/wt or KO/KO relative to wt.

DMC1 LMMs were fitted separately for each of six prophase I substages (leptotene, early zygotene, zygotene, late zygotene, early pachytene, pachytene). MLH3 LMMs were fitted on pachytene cells only, with two responses: (i) total MLH3 foci per nucleus, and (ii) number of bivalents lacking an MLH3 focus per nucleus. Full LMM output (coefficients, standard errors, z statistics, 95% confidence intervals, and uncorrected p-values; random-effect variance components; per-mouse descriptive statistics) is reported in Statistics S1 and Table S5.
